# Group arts interventions for depression and anxiety among older adults: a systematic review and meta-analysis

**DOI:** 10.1038/s44220-024-00368-1

**Published:** 2025-03-05

**Authors:** Elizabeth A. Quinn, Emma Millard, Janelle M. Jones

**Affiliations:** 1 Centre for Brain and Behaviour, Department of Psychology, School of Biological and Behavioural Sciences, Queen Mary University of London, London, UK; 2 Centre for Psychiatry and Mental Health, Unit for Social and Community Psychiatry, Wolfson Institute of Population Health, Queen Mary University of London, London, UK

**Keywords:** Psychology, Psychology

## Abstract

In this systematic review and meta-analysis, we assessed the efficacy of group arts interventions, where individuals engage together in a shared artistic experience (for example, dance or painting), for reducing depression and anxiety among older adults (> 55 yr without dementia). Fifty controlled studies were identified via electronic databases searched to February 2024 (randomised: 42, non-randomised: 8). Thirty-nine studies were included. Thirty-six studies investigated the impact of group arts interventions on depression (*n* = 3,360) and ten studies investigated anxiety (*n* = 949). Subgroup analyses assessed whether participant, contextual, intervention and study characteristics moderated the intervention–outcome relationship. Risk of bias was assessed with appropriate tools (RoB-2, ROBINS-1). Group arts interventions were associated with a moderate reduction in depression (Cohen’s *d* = 0.70, 95% confidence interval (CI) = 0.54–0.87, *P* < 0.001) and a moderate reduction in anxiety (*d* = 0.76, 95% CI = 0.37–1.52, *P* < 0.001), although there was publication bias in the depression studies. After a trim and fill adjustment, the effect for depression remained (*d* = 0.42; CI = 0.35–0.50; *P* < 0.001). Context moderated this effect: There was a greater reduction in depression when group arts interventions were delivered in care homes (*d* = 1.07, 95% CI = 0.72–1.42, *P* < 0.001) relative to the community (*d* = 0.51, 95% CI = 0.32–0.70, *P* < 0.001). Findings indicate that group arts are an effective intervention for addressing depression and anxiety among older adults.

## Abstract (web summary)

In this systematic review and meta-analysis of group arts interventions for older adults, the authors found that participation in shared artistic experience was associated with lower levels of depression and anxiety.

## Main

Depression and anxiety are major global health concerns^1^. Depression (characterized by low mood, sadness, weight changes, fatigue, worthlessness, inattention and suicidal ideation) and anxiety (characterized by psychological and physical feelings of fear and tension) are experienced by 7.0% and 3.8% of older adults (>60 yr) worldwide, respectively^2^. The first line of treatment is usually to prescribe antidepressant medications or talking therapies^3,4^. However, as older adults are at greater risk of side-effects from antidepressant medication and face barriers to accessing talking therapies (for example, treatment costs, discomfort in discussing mental health and ageist attitudes of medical staff)^5,6^, alternative evidence-based methods of supporting older adults should also be considered. Bringing together established research on the benefits of the arts and the power of groups for health^7–10^, this meta-analysis examined the evidence for a promising alternative for addressing depression and anxiety among older adults—that is, group arts interventions.

## Understanding the arts and its interventions

The arts can be grouped into five different forms: performing arts (for example, music and dance), visual arts (for example, painting and sculpture), literature (for example, creative writing), culture (for example, attending museums and concerts), and digital and film (for example, animation and film-making)^11^. Across these art forms, there is scope for either active engagement (where individuals directly participate in the artistic process—for example, painting a picture) or receptive engagement (where individuals appreciate aspects of the artistic process and/or consume art produced by other people—for example, listening to music)^12^. The diversity of art forms and modes of engagement allows for flexibility in arts interventions as individuals can take part in one or more types of arts activities concurrently or over time. Arts activities can also offer a range of auditory, tactile and visual experiences as well as provide different avenues of expression for individuals with varied interests and requirements^7^.

There is mounting evidence for the benefits of arts engagement and interventions for mental health in various populations, including older adults^13–17^. Creative and cultural participation was identified as the greatest contributor to the well-being of older adults in later life across 40 personal, social, health, resource and local indicators^18^, and a review of available research suggests that arts interventions are cost-effective solutions that support mental health and reduce burden on health and social care services^19^. Although this evidence is encouraging, there have been mixed findings across recent systematic reviews on the impacts of the arts on the mental health of older adults^20,21^. The lack of clarity across these reviews may stem from the wide variation in the characteristics of participants (for example, age), contexts (for example, setting and country), intervention (for example, art type, intervention type and intervention dose) and study design (for example, control group type) in this literature (ref. ^22^ contains details of the INNATE framework, which synthesizes and offers a comprehensive discussion of the 139 elements that encompass these broad characteristics). To better understand when and for whom arts interventions can benefit mental health, in this meta-analysis we considered whether the most commonly reported aspects of these four characteristics might moderate the impact(s) of arts interventions on depression and anxiety for older adults.

## Participant characteristics

### Age

Arts interventions delivered to a wide range of age groups have been associated with improved mental health (for example, refs. ^23,24^). However, age has not typically been considered as a potential moderator of this relationship for older adults despite evidence that circumstances that can trigger poor mental health shift with age^25^. Given that many of the risk factors for poor mental health in later life—such as the reduction of daily activities, loss of a spouse or increased physical illness—increase with age^26,27^, it is important to assess the potential impact(s) of arts interventions across the ‘younger old’ to ‘older old’ age spectrum.

## Contextual characteristics

### Setting

Arts interventions for older adults are often delivered in the community via local arts programs or charities, or in care homes. As older adults living in the community have to get to the intervention, whereas care-home residents have the intervention delivered in their homes, there may be differences in adherence or the intervention dose received that influences efficacy. The benefits of arts interventions in the community versus care homes may also differ because a higher proportion of care-home residents experience depression and anxiety relative to older adults living in the community^6,26^. In light of these potential differences, we investigated whether setting influenced the intervention–outcome relationship.

### Country

Although depression and anxiety are global issues, research tends to focus on wealthier western countries despite the fact that more than 70% of the mental health burden falls within low- and middle-income countries (LMICs; for example, China and India) compared with high-income countries (HICs; for example, the United States and United Kingdom)^28,29^. There is also a disparity in the resources available for treating mental health conditions between HICs and LMICs, which highlights the importance of examining the potential impact(s) of arts interventions in resource-rich and resource-poor countries.

## Intervention characteristics

### Art type

The scant research comparing different art types suggests that there is no differential impact on mental health^30,31^. There has been little investigation into whether the number of art types within an intervention might impact the health and well-being of older adults. Examining whether the art type or their number influences the intervention–outcome relationship can clarify whether certain activities or their combinations should be recommended to support the mental health of older adults.

### Intervention type

Arts interventions may be classified as creative arts therapy (for example, music therapy and dramatherapy), led by a trained therapist with arts conducted in a therapeutic context^32^, or arts activities, led by artists/volunteers and designed for non-therapeutic contexts. This distinction is noteworthy because these interventions have different goals: creative arts therapy seeks to improve mental health by using the arts to understand and communicate difficult thoughts and feelings, whereas arts activities encourage engagement, either actively or receptively, to learn, experience, experiment or create something specific^33,34^. Given a recent systematic literature review suggesting that arts therapy interventions are related to better outcomes for older adults than arts activity interventions^20^, we sought to clarify the impact of arts therapy versus arts activity on depression and anxiety for older adults.

### Intervention dose

Interventions can differ in the number of sessions provided (for example, single or multiple sessions), session duration (for example, minutes and/or hours per session), session frequency (for example, once or more per week/month) and duration of the intervention (for example, one day to several months). These differences in intervention dose have been found to impact mental health outcomes^35^, although the mixed evidence suggests that longer interventions can both reduce depression^36^ and provide a barrier to attendance that impairs benefits^37^. In considering these intervention characteristics, we may clarify whether a particular dose is needed to support the mental health of older adults.

## Study design characteristics

### Control group type

Different types of control groups are used in randomized control and non-randomized controlled studies^38^. These include usual activity/treatment (participants continue their day-to-day activities), waiting list (participants are placed on a waiting list and receive the intervention after its completion by the intervention group), active (participants are offered another activity to control for other potential treatment effects) and placebo (participants are given a treatment/intervention without therapeutic impact) controls. Control group type has been found to impact effect sizes in meta-analyses exploring depression treatments^39^, with waitlist and/or usual activities control groups inflating effect sizes relative to placebos when compared with cognitive behavioral therapy (CBT) interventions^40,41^. We sought to clarify whether the control group type matters for detecting the effectiveness of group arts interventions on depression and anxiety for older adults.

## The enhanced benefits of group-based interventions

An important component of interventions is whether they are administered individually, where participants engage in an activity alone (for example, meditation or individual music listening), or in groups, where participants engage in activities with other people (for example, a weekly walking club or peer support group). According to the social identity approach to health^8,9^, groups may be a particularly effective way to deliver interventions because they afford opportunities to share experiences with peers, to (re)gain a sense of identity and to feel a sense of connectedness and belonging, which help individuals to access the tangible and intangible resources from other group members (for example, advice, information and comfort) that are needed to buffer against stress and promote health^8,9,42–45^. An extensive body of research drawing on this approach demonstrates the benefits of group-based interventions for mental health and well-being for older adults and in the context of the arts (for example, refs. ^30,46–51^). Group arts interventions may therefore reflect an effective way of reducing depression and anxiety in older adults.

This meta-analysis synthesized the available evidence to determine whether group arts interventions were effective at reducing depression and anxiety among older adults. We focused on group arts interventions given the noted benefits of group-based delivery as well as their cost-effectiveness and potential for scalability. We took a systematic approach in identifying controlled studies (that is, randomized control trials and quasi-experimental studies with control groups)^52^ to investigate the strength of the effect and assess whether participant, contextual, intervention and study characteristics might influence the intervention–outcome relationship via subgroup analyses and meta-regressions. This meta-analysis moves beyond recent literature reviews (for example, ref. ^20^) and provides critical and novel insights relative to recent meta-analyses in this area (for example, ref. ^53^—we included multiple art types and focused on mental health— and ref. ^54^—we compared arts therapy with arts activity interventions and focused on older adults without dementia).

## Results

### Study selection and inclusion

**Fig. 1:**
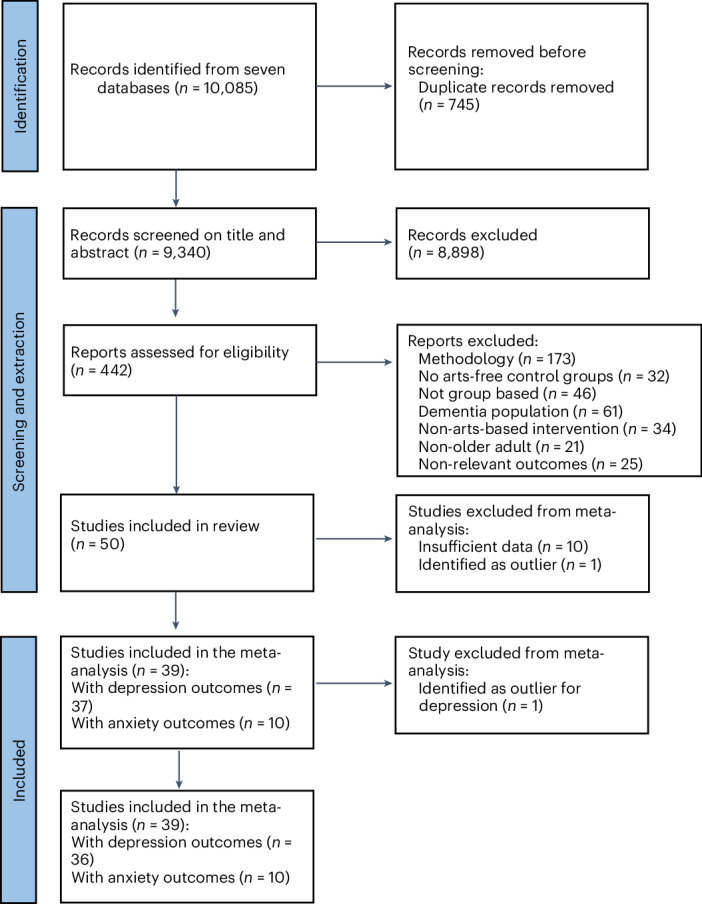
Study selection process. Preferred Reporting Items for Systematic reviews and Meta-Analyses (PRISMA) 2020 flowchart.

Removal of duplicates yielded 9,340 studies. On completion of title, abstract and full-text screening, 50 studies were deemed eligible for inclusion (Fig. 1), all of which were included in the study summary (47 reported depression outcomes and 14 reported anxiety outcomes; Appendix A of Supplementary Information). A complete reference list of the included studies can be found in Appendix B of Supplementary Information.

Ten studies^31,55–63^ comprising nine depression outcomes and three anxiety outcomes could not be included in the meta-analysis as they did not report the data needed to calculate the effect size and due to non-response or lack of contact details for the authors. Initial analyses indicated that the effect sizes from one study^64^ were extremely high (depression, Cohen’s *d* (or effect size; *d*) = 26.42; anxiety, *d* = 33.26). After looking at the forest plots, one further study^65^ was identified as an outlier on depression (*d* = 3.13). These studies were also removed from the analyses. This left 39 studies^16,17,58,65,66–101^, which reported 36 depression outcomes and ten anxiety outcomes, for entry into the meta-analysis.

### Summary of the characteristics of the included studies

#### Participant characteristics

Of the 50 included studies, all but three presented the average age of their participants, which ranged from 64.75 to 84.04 yr. The average participant age fell into a young-old category (that is, 65–74 years old) in more than half the studies (*n* = 28) and into a middle-old category (that is, 75–84 years old) in the remaining studies (*n* = 22). As expected given the longer lifespan of women, the participants were predominantly female in the majority of studies: 75–100% female participants in *n* = 28 studies and 50–74% female participants in *n* = 15 studies. A minority of studies had <50% female participants (*n* = 4). Three studies did not provide gender details for the participants. Most studies did not present information about participant ethnicity or race (*n* = 36). Of the nine studies that did include data on participant race, six had over 90% white participants. Three studies mentioned a mix of racial categories: the first study included 11.1% Black, 4.4% Hispanic, 9.9% Asian and 75.6% white participants; the second included 26% non-Hispanic Black, 20% Asian, 18.5% Hispanic and 35% non-Hispanic white participants and the third included 4% Native American, 24% African American, 48% white, 8% mixed ancestry and 16% not reported.

Of the five studies that included information on the ethnicity of the participants, one had 100% African American participants, one had 100% Korean American participants, one had 100% Indonesian, one had 100% European American and one had 100% Chinese participants.

#### Contextual characteristics

The majority of arts intervention studies were conducted in the community (*n* = 32) and care homes (*n* = 15). One study comprised participants from a mix of community and care homes^55^ and a small number of US-based studies were conducted in senior retirement communities, which the authors felt should be categorized separately to care homes due to the differences in on-site amenities, activities and independence (*n* = 2). The studies were conducted across 21 countries, highlighting the international interest in the impact of group arts interventions on mental health in later life. Most studies were conducted in the United States (*n* = 12), followed by China (*n* = 9); Taiwan and Canada (*n* = 3 each); Brazil, Iran, Singapore, Tanzania, Turkey and the United Kingdom (*n* = 2 each); and the Czech Republic, Greece, Indonesia, Ireland, Malaysia, the Philippines, Portugal, South Korea, Thailand, France and Denmark (*n* = 1 each). In terms of income, nine of these countries can be characterized as LMICs and 12 as HICs^102^.

#### Intervention characteristics

The majority of interventions were structured around one art type. The three most common art types reported were dance (*n* = 17), music (*n* = 12) and visual arts (*n* = 11). The remaining single art-type interventions used creative writing (*n* = 3) or drama (*n* = 1). One intervention compared two separate arts interventions (visual arts versus music interventions) with a control group. Five of the interventions combined multiple art types including music and movement; music, imagery and movement; visual arts and drama; visual arts and storytelling; and drama (performing magic) and visual arts (*n* = 1 each). There were over three times the number of arts activity interventions (*n* = 39) relative to arts therapy interventions (*n* = 11).

All studies reported the intervention duration and all but two studies reported the number of sessions in the intervention. The duration of the interventions ranged from four to 96 weeks, with the majority of interventions lasting 4–12 weeks (*n* = 27). The total number of sessions ranged from six to 96, with half of the interventions including 6–12 sessions (*n* = 25).

#### Study design characteristics

The vast majority of the 50 included studies randomly assigned participants into intervention and control groups (*n* = 42). The remaining studies did not use randomization to determine intervention and control group assignment (*n* = 8). The majority of studies compared arts interventions with usual activity control groups (*n* = 25), whereas the other studies used a waiting-list (*n* = 7) or an active (*n* = 12) control group. Two studies indicated that they used controls but did not report the type of control group used. Two studies compared an arts intervention with two control groups: one active and one usual activities. Two studies did not provide any control group information.

### Depression

**Fig. 2:**
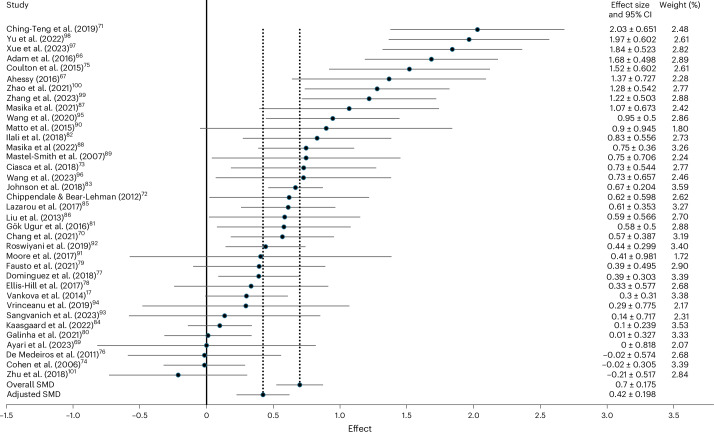
Effect of group arts intervention on depression. Forest plot including 36 studies (*n* = 3,360 total participants)^17,66–101^. At zero (solid line) there is no difference between the intervention and control conditions, positive values indicate a positive effect of the intervention (that is, reducing depression) and negative values indicate that there is a negative effect of the intervention (that is, increasing depression). The standardized mean differences (*d*) are plotted (black dots): 0–0.2, no/negligible effects; 0.21–0.49, small effects; 0.50–0.79, moderate effects; and >0.8, large effects. The overall standardized mean difference (SMD) reflects the average effect across all included studies (*d* = 0.70). The adjusted SMD reflects the average effect across all included studies after adjusting for publication bias using Duval and Tweedie’s trim-and-fill procedure^104^ (*d* = 0.42). The effect and its 95% CI are reported (horizontal line through each black dot, specific values to the right of the visualization) along with the weight given to the finding within the meta-analysis. Weights are from a random-effects model.

The initial analysis was based on 37 studies evaluating the effect of group arts interventions on depression scores in older adults (*n* = 3,379). As noted earlier, one outlier was identified following the initial calculation and examination of the forest plot^65^. This reduced the number of studies to 36 and the number of participants (*n* = 3,360 total; *n* = 1,769 intervention and *n* = 1,591 control). Results indicated a significant moderate effect (*d* = 0.70, 95% confidence interval (CI) = 0.52–0.87; *P* < 0.001, heterogeneity statistic *I*^2^ = 0.81%; forest plot in Fig. 2, sensitivity analysis in Table 1 and discussion of heterogeneity in Methods).

**Table 1:** Sensitivity analyses for depression

	Outlier included	Outlier removed	Non-randomized studies removed*	MCI studies removed*	Adjusted for publication bias**
Overall effect size and 95% CI	*n* = 37 *d* = 0.73 95% CI = 0.55–0.91	*n* = 36 *d* = 0.70 95% CI = 0.52–0.87	*n* = 32 *d* = 0.67 95% CI = 0.49–0.85	*n* = 25 *d* = 0.71 95% CI = 0.49–0.93	*n* = 36 *d* = 0.42 95% CI = 0.35–0.50
Egger’s test *P* value	0.004	0.010	0.020	0.011	Not applicable

*Performed with outlier removed.

**Duval and Tweedie trim and fill^104^.

Two-tailed tests were performed.

#### Publication and reporting bias

**Fig. 3:**
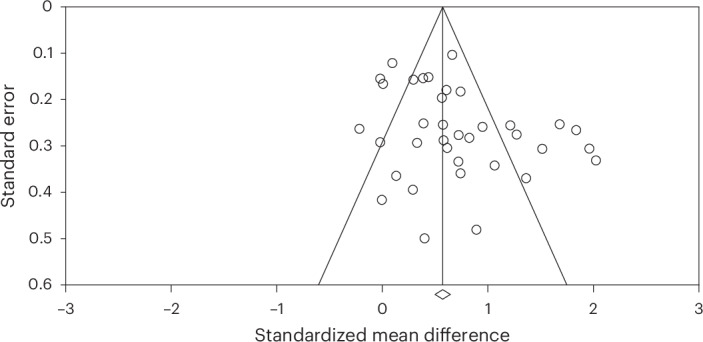
Publication bias in studies of the effects of group arts intervention on depression. Each of the included studies is represented by an unshaded circle in the funnel plot. This unshaded circle reflects the standardized mean difference associated with the study. The overall effect is represented by the central line demarcated with a diamond on the *x* axis. The left pyramid line represents the lower 95% CI. The right pyramid line represents the higher 95% CI. The standard error on the *y* axis represents the study precision. Studies toward the top of the pyramid are larger studies with greater precision. Studies toward the bottom of the pyramid are smaller studies with less precision. Bias and heterogeneity are indicated by a lack of symmetry in the distribution of the studies across the pyramid (for example, falling outside of the CIs, favoring one side of the distribution over the other).

There was evidence of publication bias (Egger’s test, *P* = 0.010; funnel plot in Fig. 3). A Duval and Tweedie’s trim-and-fill procedure adjusted for this bias by imputing ‘missing’ studies from the funnel plot and producing an effect size that accounts for funnel plot asymmetry^103,104^. After this adjustment, the effect size fell from moderate to small (*d* = 0.42, 95% CI = 0.35–0.50, *P* < 0.001; funnel plot with the imputed studies in Supplementary Information, Appendix C).

#### Subgroup analyses

Subgroup analyses were run on study setting, country, art type, intervention type and control group type. There were insufficient multimodal interventions to explore differences in single and multimodal arts interventions for either depression or anxiety. All 36 depression outcomes were included for intervention type (arts activity and arts therapy) and country (LMIC and HIC) analyses. As there were not enough of the supported living studies (*n* = 2) to be included^72,76^, the study setting analyses compared participants living in the community and care homes only. The art-type analyses compared visual arts, music and dance only as there were insufficient studies investigating writing or drama for inclusion^72,76,90^. The two interventions that used more than one art type were also excluded from this analysis^91,96^. Two studies were removed from the control group analyses as not enough information was presented to define the control group type^74,86^.

##### Study setting

A significant between-group effect (*P* = 0.005) was found between community (*d* = 0.51, 95% CI = 0.32–0.70, *P* < 0.001) and care-home (*d* = 1.07, 95% CI = 0.72–1.42; *P* < 0.001) settings, with a stronger effect found for participants in care homes.

There were no differences between subgroups for art type, intervention type, country or control group type (Supplementary Information, Appendix D).

In addition to the planned analyses, exploratory subgroup analyses on average baseline depression scores found a marginal between-group effect (*P* = 0.063) between participants who experienced any depression (*d* = 0.81, 95% CI = 0.56–1.06, *P* < 0.001) and participants who experienced no depression (*d* = 0.49, 95% CI = 0.27–0.71, *P* < 0.001), which suggests a stronger effect of group arts interventions for participants who experienced any depression (Supplementary Information, Appendix D).

#### Meta-regression

We explored the association between the depression effect sizes and various continuous moderators—that is, the average participant age, intervention duration (weeks), session length (min), total number of sessions and intervention intensity (time (min) per week). No significant associations were found (*P* > 0.05 for all comparisons; Supplementary Information, Appendix E).

### Anxiety

**Fig. 4:**
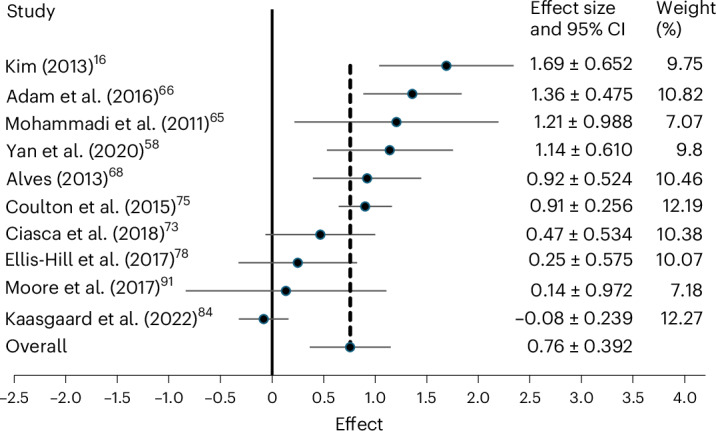
Effect of group arts intervention on anxiety. Forest plot of ten studies (*n* = 949 total participants)^16,58,65,66,68,73,75,78,84,90^. At zero (solid line) there is no difference between the intervention and control conditions, positive values indicate a positive effect of the intervention (that is, reducing anxiety) and negative values indicate that there is a negative effect of the intervention (that is, increasing anxiety). The standardized mean differences (*d*) are plotted (black dots): 0–0.2, no/negligible effects; 0.21–0.49, small effects; 0.50–0.79, moderate effects; and >0.8, large effects. The overall SMD reflects the average effect across all included studies (*d* = 0.76). The effect and its 95% CI are reported (horizontal line through each black dot, specific values to the right of the visualization) along with the weight given to the finding within the meta-analysis. Weights are from a random-effects model.

This analysis was based on ten studies evaluating the effect of group arts interventions on anxiety scores in older adults (*n* = 949). The results indicated a moderate effect: anxiety symptoms were significantly improved in older adults who engaged in a group arts intervention (*n* = 487) compared with those who did not (*n* = 462, *d* = 0.76, 95% CI = 0.37–1.52, *P* < 0.001, *I*^2^ = 0.85%; forest plot in Fig. 4, sensitivity analyses in Table 2 and discussion of heterogeneity in Methods).

**Table 2:** Sensitivity analyses for anxiety

	Total anxiety studies	Non-randomized and MCI studies removed*
Overall effect size and 95% CI	*n* = 10 *d* = 0.76 95% CI = 0.37–1.52	*n* = 8 *d* = 0.63 95% CI = 0.22–1.05
Egger’s test *P* value	0.125	0.217

*The same two studies contained MCI populations and non-randomized control group.

Two-tailed tests were performed.

#### Publication and reporting bias

**Fig. 5:**
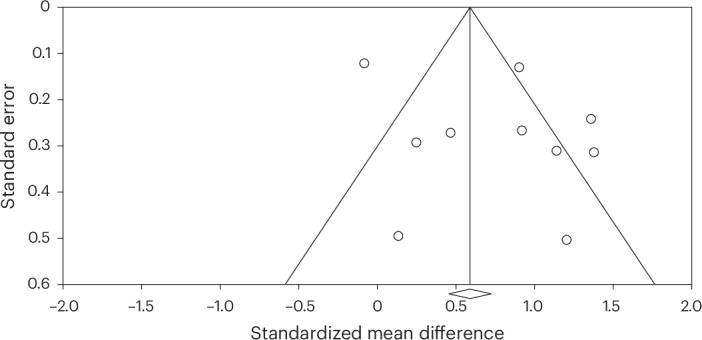
Publication bias in studies of the effect of group arts intervention on anxiety. Each of the included studies is represented by an unshaded circle. This unshaded circle reflects the standardized mean difference associated with the study. The overall effect is represented by the central line demarcated with a diamond on the *x* axis. The left pyramid line represents the lower 95% CI. The right pyramid line represents the higher 95% CI. The standard error on the *y* axis represents study precision. Studies toward the top of the pyramid are larger studies with greater precision. Studies toward the bottom of the pyramid are smaller studies with less precision. Bias and heterogeneity are indicated by a lack of symmetry in the distribution of the studies across the pyramid (for example, falling outside of the CIs, favoring one side of the distribution over the other).

There was no evidence of publication bias (Egger’s test, *P* = 0.125; funnel plot in Fig. 5).

#### Subgroup analyses

As there were only ten studies, only intervention type (arts activity and arts therapy) and country (LMIC and HIC) could be explored. All ten studies were included in both these analyses and no significant results were found (*P* > 0.05 for all comparisons; Supplementary Information, Appendix F).

#### Meta-regression

We explored the association between the anxiety effect sizes and continuous moderators—that is, average participant age, intervention duration (weeks), session length (min), total number of sessions and intervention intensity (time (min) per week). No significant associations were found (*P* > 0.05 for all comparisons; Supplementary Information, Appendix G).

## Discussion

Synthesizing across 39 controlled studies, this meta-analysis found that engaging in group arts interventions significantly reduced depression and anxiety symptoms relative to engaging in other non-arts-based activities, one’s usual activities only or remaining on a waiting list for arts interventions. Critically, the observed effect sizes were comparable to effect sizes found in other meta-analyses of controlled studies exploring pharmacological, therapeutic and activity-based treatments for depression and anxiety in later life^3,4,105–109^. We provide evidence that group arts interventions can reduce depression and anxiety symptoms among older adults and lend further support to the growing literature on the benefits of group-based interventions for health and well-being^30,46–51^.

This Analysis also makes an important contribution through its systematic examination of the moderating effects of participant, contextual, intervention and study characteristics on depression and anxiety in later life. Regarding contextual characteristics, group arts interventions were more effective at reducing depression when delivered in care homes relative to community settings, possibly reflecting a reduction in commonly cited barriers to attendance and engagement^110,111^, which may allow for a higher ‘dose’ of the intervention compared with other settings and, in turn, provide the time, interactions and activity needed to foster mental health^35,112,113^. Findings might also reflect differences in depression severity for individuals living in care homes or the community^6,26^. Of the included studies, 50% of community samples were classified as experiencing no depression. However, where depression was experienced, its classification was largely similar for community and care-home samples (that is, mild). As exploratory analyses suggested that depression symptoms were reduced for both older adults experiencing no depression or any depression—with marginally stronger effects for older adults experiencing any depression—it may be that group arts interventions provide a universal benefit that is particularly helpful for the individuals who need them most (that is, individuals living in care homes and experiencing any depression)^19^.

There were no moderating effects of the other participant, contextual, intervention or study design characteristics on the intervention–outcome relationship. The average age of the participants did not moderate either depression or anxiety, indicating that group arts interventions can support mental health for young and old alike. The benefits of group arts interventions may therefore be leveraged to support mental health across an individual’s lifespan^7–9,22,32^. That group arts interventions were equally as effective at reducing depression and anxiety across LMICs and HICs suggests that they may be of universal benefit to older adults regardless of the mental health provision in their country. The innovation and creativity of group arts interventions may be generated by relying on the resources that individuals have at hand rather than being inaccessible by nature or design. No difference was found across art type (that is, visual arts, dance and music), in line with previous research that group arts interventions can support mental health irrespective of what people do^30^. For intervention characteristics, similar to previous reviews^20,21^, drama, creative writing and other art types were under-represented in the literature. We join these authors in calling for more research on these under-represented art types to better understand whether they may benefit the mental health of older adults. There were no differences in the impacts of arts therapy and arts activity interventions—both reduced depression and anxiety for older adults. Group arts interventions also reduced depression and anxiety regardless of intervention or session length. Additional research into intervention characteristics may be needed to ensure that healthcare providers as well as arts and health practitioners find the right balance between available resources and the amount of time and number of sessions needed to deliver interventions that benefit the mental health of older adults.

In terms of study characteristics, we did not observe an effect for control group type (active, waiting list or usual activity) as a moderator. Although we can conclude that group arts interventions work relative to control groups in general, more research is needed to determine whether this effect persists relative to comparable activities in particular.

Although our overall findings are meaningful, they must be interpreted in the context of the limitations of the meta-analysis. One limitation is that there was evidence of publication bias in the depression outcomes, suggesting that null or negative findings are probably missing from the published literature. We ensured the robustness of this meta-analysis by assessing risk of bias for included papers using RoB 2 and ROBINS1 (which are considered the gold-standard instruments to evaluate quality and presence of bias in randomized control and non-randomized controlled trials) by conducting Egger’s test, running sensitivity analyses and correcting for bias using the trim-and-fill procedure, which reduced the magnitude but did not eliminate the observed effect for group arts intervention on depression^103,104,114^. We would call on researchers to publish their negative and null results via archives or through open science pages to increase their accessibility and chances for inclusion in meta-analyses of the literature. We would also call on academic journals to provide clearer channels for publishing null and negative effects. We are particularly encouraged by the increase in options for registered reports, which should help in this regard over time.

A second limitation is the high study heterogeneity, which suggests that the included interventions tended to differ from each other on several dimensions^115^. This was expected given the diversity of the arts^22^ and does not diminish the overall observed effects. The prediction interval indicated that the vast majority of participants would gain at least some benefit from engagement in group arts interventions, despite the wide range in effect sizes for both depression and anxiety (Supplementary Information, Appendixes J and K). Heterogeneity might have arisen from the measures used to assess depression and anxiety. Although these were all validated, standardized scales, they were largely self-administered and differed across studies. Heterogeneity might also be due to differences in the elements of the delivered interventions. One benefit of performing moderation analyses is that the potential causes of heterogeneity can be explored. Our results highlighted that setting (care home or community) contributed to some of this variation. Future research should explore which factors enhance the consistency of the observed effects. The idea of standardizing group arts interventions may be unpalatable to some but this does not have to take away from the aesthetic and creative nature of the interventions or the enjoyment gained from taking part in the creative activity. Understanding and designing interventions that minimize heterogeneity will help to harness the effective elements of group arts interventions and maximize their impacts on depression and anxiety.

A third limitation is that this review did not include individual arts interventions and was therefore unable to compare the relative benefits of individual versus group arts interventions for depression and anxiety. Evidence from social psychology suggests that group-based activity interventions are more impactful at supporting mental health compared with individual-based activity interventions^116,117^. Evidence from the arts and health literature suggests that both individual and group arts interventions are associated with reductions in depression and anxiety but has tended not to examine their relative effects^20^. Furthermore, evidence from psychotherapy research is mixed, with evidence of a small short-term advantage in individual CBT for depression^118^ and no difference between individual and group CBT or individual and group therapy on depression and anxiety^119,120^. If future meta-analyses find that group arts interventions are equivalent to, or more effective than, individual arts interventions, this will further support their use as a cost-effective and time-saving option that can support the mental health of several older adults at once.

Together, our findings suggest a universal benefit of group arts interventions across different characteristics, including art type (that is, visual arts, music and dance), for addressing depression and anxiety. This has practical implications for the mental health of older adults. Research has shown that receipt of a preferred psychosocial intervention is associated with lower dropout in mental health services^121^. Moreover, the National Institute for Health and Care Excellence guidelines for treating depression suggest that, where possible, patients should be involved in choosing which recommended treatment(s) they prefer^3^. If offering group arts interventions, providing individuals with a choice in what they do, may enhance engagement and therefore, the potential for well-being (evidence of the importance of program variety in ref. ^122^). Our findings are also relevant for social prescribing initiatives designed to help older adults who need support with their mental health, are socially isolated or disenfranchised, or with one or more long-term health conditions^123^. Social prescribing enables general practitioners to refer individuals to community activities (including arts activities) alongside other treatments to help to support the health and well-being of their patients^124^. Although arts interventions form a key part of the social prescribing initiative, there has been a lack of clarity on whether they should be recommended for mental health. Our findings support the continued use of group arts interventions as part of social prescribing initiatives and provide concrete evidence that health advisory bodies such as the National Institute for Health and Care Excellence should include group arts interventions alongside their other recommendations for addressing depression and anxiety in older adults.

## Methods

### Transparency and openness

We adhered to the PRISMA 2020 guidelines for systematic reviews^125^. Data were modeled and visualized using Comprehensive Meta-Analysis (CMA) version 3 (ref. ^126^). The study protocol was preregistered on PROSPERO (https://www.crd.york.ac.uk/prospero/display_record.php?RecordID=176701; ID CRD42020176701). Because this review only involved the use of secondary anonymized data from other research studies, it did not require ethical approval.

### Search strategy

The search strategy was developed by the three members of the study team (a PhD student with a background in drama and psychology (E.A.Q.), an accredited music therapist (E.M.) and a social psychologist (J.M.J.)). The initial search took place between 8 and 15 May 2020 using the following databases: Cochrane Library, PsycARTICLES, PsycINFO, EMBASE, Web of Science, PubMed and Google Scholar. This search was extended using the same databases twice (14–28 February 2022 and 22–29 February 2024). Gray literature and unpublished work were sought by placing calls for literature on relevant academic listservs (European Association of Social Psychology, The Society for Personality and Social Psychology and British Society of Gerontology). The full search terms can be found in Appendix H of the Supplementary Information.

### Study selection criteria

#### Participants

To be included, the average age of the study participants was set at ≥55 yr. This minimum age was set to account for different understandings of old age across industrialized and developing countries^127^. As we were interested in understanding the potential benefits of arts interventions for older adults without impairment in cognitive functioning that affects daily life, populations of individuals with dementia were excluded. However, studies looking at older adults with MCI were included as MCI does not necessarily lead to dementia or impact functioning in daily life^128^.

#### Intervention

In light of our focus on group-based interventions, interventions delivered individually were excluded. The intervention could include either active or receptive arts engagement; however, the focus of intervention had to involve a specific art type (for example, an intervention where participants did yoga while listening to music would be excluded). To be included, interventions had to involve engagement in one or more creative arts activities or therapies (for example, music, dance, visual arts, drama and creative writing). For this review, we categorized arts therapy interventions and arts activity interventions using the British Association of Music Therapy definition, which stresses the importance of the therapeutic relationship that develops between therapist and client(s) and therefore requires that the intervention is led by a trained therapist to be classed as arts therapies^129^. If the qualifications of the facilitators were unclear, the intervention was categorized as an arts activity intervention, even if the intervention was described as art therapy in the study (*n* = 3). Decisions on whether interventions were categorized as an art therapy or art activity were made by E.A.Q. and E.M.

#### Comparison

Only interventions with control groups were included. Control groups could include treatment as normal, a waiting list or active control groups where participants took part in a non-art-related activity.

#### Outcome measures

Studies that measured depression and anxiety pre- and post intervention were included. For anxiety, trait-specific measures such as the trait items from the state–trait anxiety inventory^130^ were not included as they measure a stable dimension of personality, which is unlikely to be affected by engaging in an intervention^131^. All included studies used standard validated measures of depression and anxiety that are typically used in clinical settings (details of the measure(s) used in each study in Supplementary Information). Of the studies included in the meta-analysis, the average baseline depression scores were ‘normal’ for 13 studies, ‘mild’ for 17 studies, ‘moderate’ for four studies, and ‘severe’ for two studies. The average baseline anxiety scores were normal for five studies, mild for three studies, moderate for one study and severe for one study. As noted in our pre-registration, the search included several well-being outcomes in addition to depression and anxiety. However, in this Analysis, we have chosen to focus on depression and anxiety only for parsimony.

#### Study design

Both randomized control and non-randomized controlled trials were included to capture a wide range of interventions and approaches.

#### Other

All studies had to be written in English to be included.

### Study selection and data extraction

The studies identified through this search were screened first by title and abstract and then by reading the full text. E.A.Q. screened 100% and E.M. screened 25% of the papers at these stages. Data from the included studies were extracted into an Excel spreadsheet by E.A.Q. (100%) and E.M. (15%). Extracted data were: country of study, number of participants, study setting (care home or community), average participant age, participant gender, participant ethnic background, MCI status, study methodology, intervention art type(s), number of intervention art types, engagement type (active/receptive), nature of intervention (art therapy/arts activity), control group type, intervention length and measures of depression and/or anxiety. Any disagreements or queries were discussed and resolved by J.M.J.

### Risk-of-bias assessments

Risk of bias was assessed using the Cochrane risk-of-bias tool RoB 2 (ref. ^132^) for the randomized controlled trials and ROBINS-I^133^ for the non-randomized controlled studies. According to the RoB 2 assessment, risk of bias in randomized control trials is categorized as low risk, some concern or high risk. According to the ROBINS-I assessment, risk of bias in non-randomized controlled studies is categorized as low, moderate, serious, critical or no information. Results from these assessments are summarized in the Results section. Studies were not excluded on the basis of risk-of-bias results.

### Method of synthesis

Data were transferred from the extraction sheet to CMA version 3 (ref. ^126^). Pre- and post-test means and s.d. values were used to compare individuals who had received a group arts intervention to individuals who had not. If these data were unavailable, *t*-test scores, mean change *M*_∆_ scores and s.d. values or Cohen’s *d* and 95% CIs were used. The CMA software was then used to generate the overall effect size. Forest plots were created using Excel for MS Office^134^.

The mean and s.d. values from studies that contained two or more relevant intervention groups or two or more relevant control groups were aggregated together^135^. Similarly, the effect sizes and s.e. from studies that used more than one measure for a single outcome (for example, measuring depression with both Beck’s Depression Inventory (BDI) and the Geriatric Depression Scale (GDS)) were aggregated together to form a single score^135^.

### Statistical analysis

As high levels of heterogeneity were expected in the observed effects due to the range and variation of key features across studies (that is, intervention classification, setting, country and participants), a random-effects meta-analysis was used^136^. A standardized mean difference (Cohen’s *d*) and s.e. was calculated for each individual study and then pooled to create an overall effect size. The precision of this effect size was estimated using 95% CI values (calculation formulas in ref. ^137^). Cohen’s rule of thumb was used when interpreting effect sizes so that *d* values of 0–0.2 were considered negligible, *d* > 0.2 was considered a small effect, *d* > 0.5 was considered a moderate effect and *d* > 0.8 was considered a strong effect^138^. Positive effect size values indicate a reduction in depression/anxiety symptoms.

Sensitivity analyses were conducted on potential outliers, non-randomized studies and studies with MCI samples. This was done by removing these studies from the analysis to see the extent to which the overall effect changed. To avoid publication bias, the authors of studies with unreported outcomes were contacted to try and obtain these data. Publication bias was assessed using Egger’s test^139^ for small study effects and examining funnel plots for asymmetry. Egger’s tests and funnel plots were created using CMA. A significant Egger’s test indicated potential publication bias.

Heterogeneity was explored in accordance with the guidelines from Borenstein and colleagues^140^. The *Q* statistic tests the null hypothesis of no heterogeneity to explore whether the true effect is the same across all studies. A significant *Q* statistic confirms the need for a random-effects model. The *I*² statistic estimates the proportion of variation (%) that indicates meaningful differences between the intervention studies (often referred to as the true effects) that are not due to sampling error. The higher the *I*² percentage, the greater the proportion of between-study variance that is not due to sampling error.

It is recommended that the variance of the true effects is explored using the *τ*² statistic and the prediction interval if search results produce at least ten studies and there is no funnel plot asymmetry^140,141^. The *τ*² statistic indicates the variance of the true effects and *τ* the s.d. of the true effects. The *τ* statistic can estimate an approximate 95% range of the true effects by creating an interval of 1.96 τ below and above the random-effects mean, known as the prediction interval. The CMA prediction interval program was used to calculate the dispersion of the true effects of engaging in a group arts intervention on depression and anxiety symptoms in 95% of all populations comparable to those in our sample. This can be used to estimate how generalizable the results are to populations comparable to those in our Analysis and can indicate the extent to which the intervention benefits this population^142^.

Moderators were chosen and detailed in the study protocol. Categorical moderators were explored using subgroup analyses if there were ≥10 studies available for analysis and at least three studies per subgroup category^141^. Differences between subgroups were explored by performing a between-group significance test^135^. An *I*² statistic was also computed for the different subgroups to estimate the variability in effects arising from meaningful differences rather than sampling error. Meta-regression analyses were used to explore continuous moderators where a statistically significant regression coefficient indicates a linear relationship between the moderator variable and the effect size^141^. CMA was used to run these analyses.

### Risk of bias summary

In the randomized studies, few studies reported their randomization process/allocation concealment, which led to 46% being assessed as some concerns for randomization bias and the remaining 54% assessed as low risk. The reporting of deviations from the interventions was often unclear or not presented, resulting in 7% being assessed as high risk of bias due to deviations, 7% being assessed as some concerns of risk of bias due to deviations and 56% being assessed as low risk. Attrition rates were generally good—71% of studies assessed as low risk for attrition bias. No studies scored at low risk for bias in outcome measurement as these were self-assessed in all interventions. This meant that the participants were considered the assessors and, due to the nature of these intervention studies, could not be blinded to whether or not they received the intervention. Thirty-four per cent of studies were assessed as low risk for reporting bias and 64% were assessed as some concerns, mostly due to not reporting pre-registration or protocol information.

In the non-randomized studies, risk was mainly found in domains one (risk due to confounding) and six (risk in measurement of outcomes). All studies were assessed to be at high risk of bias due to confounding factors as none of the studies included all well-known confounders in their analyses. No studies were assessed as low risk for bias in the measurement of outcomes due to the subjective nature of the assessments (as found with the randomized studies). Eighty-eight per cent of studies were assessed as low risk of bias due to participant selection, 100% as low risk for classification of intervention bias and 77% as low risk for bias due to deviations from intended interventions. Fifty-five per cent of studies were assessed as low risk of bias due to both missing data and selection of reported results. This was due to a higher level of dropouts in the non-randomized interventions and, as found with the randomized studies, a lack of study pre-registration. The complete summary tables for risk of bias are in Supplementary Information, Appendix I.

### Heterogeneity summary

For depression, there was a significant amount of heterogeneity (*Q* = 180.11, df = 35, *P* < 0.001), confirming that a random-effect model was appropriate. We observed a high proportion of variance unattributable to sampling error (*I*² = 80.5%, *τ*² = 0.21, *τ* = 0.46), suggesting meaningful differences between the group arts interventions. Heterogeneity was explored further by calculating the prediction interval. For 95% of all populations comparable to those in this analysis, the true effect size would be expected to fall within the range −0.25–1.65: group arts interventions would reduce depression among the vast majority of relevant populations, with a small minority experiencing little-to-no effect (distribution of true effects plot in Supplementary Information, Appendix I).

For anxiety, there was a significant amount of heterogeneity between the group arts intervention studies (*Q* = 60.98, df = 9, *P* < 0.001). A high proportion of variance could not be attributed to sampling error, suggesting some meaningful differences between the group arts interventions (*I*² = 85.24%, *τ*² = 0.56, *τ* = 0.31). Although there were nine, rather than the recommended ten, studies^140^, as there was no evidence of publication bias, we calculated the prediction interval. For 95% of all populations comparable to those in the analysis, the true effect size would be expected to fall within the range −0.61–2.13. This indicated that group arts interventions would reduce anxiety with moderate-to-strong effects in the vast majority of relevant populations but that some harmful effects might be found in a small percentage of relevant populations (distribution of true effects plot in Supplementary Information, Appendix J). It should be noted that the heterogeneity and prediction interval for the anxiety studies greatly increased following the third search and the addition of a single study^143^ (previous results: *I*² = 52.17%, *τ*² = 0.08, *τ* = 0.28; prediction interval, 0.16–1.62). This study investigated the use of singing for lung health as well as for quality of life (including measures of depression and anxiety) in adults with chronic obstructive pulmonary disease. As this study had the highest number of participants, it was given the highest weight when calculating overall effect size and heterogeneity. It may be that the overall poor health of the participants in this study is having undue influence on heterogeneity. However, as it does not otherwise represent a statistical outlier and meets our inclusion criteria, there are no grounds for its elimination from the overall results.

### Reporting summary

Further information on research design is available in the Nature Portfolio Reporting Summary linked to this article.

## Supplementary information

Supplementary Information Supplementary Appendixes A–K.

Reporting Summary

## Data Availability

All data, analysis code and research materials (including our coding scheme) are available via the Open Science Framework (https://osf.io/h27ag/?view_only=6c25d5c7d93545ae83fd7cdf8d64609d).
