# Supplementary material for: Group arts interventions for depression and anxiety among older adults: a systematic review and meta-analysis
Source: Nat Ment Health. 2025 Mar 5;3(3):374–86. doi: 10.1038/s44220-024-00368-1 (PMC11896886; doi:10.1038/s44220-024-00368-1)

nature mental health

Supplementary information https://doi.org/10.1038/s44220-024-00368-1

Group arts interventions for depression and anxiety among older adults: a systematic review and meta-analysis

In the format provided by the authors and unedited

Supplementary Materials

Appendix A

Study Summary Table

Table 1. Characteristics of Extracted Studies

Study	Design	Outcome	Outcome Measure	Demographics	Intervention Type	Intervention length and dose	Setting Information	Art Information	Post Intervention Results Summary
**Adam et al. 2016**	Non-Randomised<br><br>Control group type= active control	Depression<br><br>Anxiety	HADS-D<br><br>HADS-A	N = 84<br><br>Mean Age = 70.89 (8.19)<br><br>Sex = 50% F<br><br>Ethnicity/Race = no info<br><br>MCI population	Non-Therapy	Length in weeks= 6<br><br>No. of sessions = 12<br><br>Length of session (mins) = 60	Intervention setting = care-home<br><br>Country = Malaysia	Engagement Type= participatory<br><br>Art Type = dance	Repeated ANOVA (No test statistics reported); Significantly lower depression ($p<.001$; $\eta^2=.35$) and anxiety ($p<.001$; $\eta^2=.29$) scores in int group compared with ctrl
**Ahessy 2016**	Randomised<br><br>Control group type: usual activities	Depression	Cornell Scale for Depression in Dementia	N = 36<br><br>Mean Age = 83.5<br><br>Sex = 86.12% F<br><br>Race = White 100%	Therapy	Length in weeks= 12<br><br>No. of sessions = 12<br><br>Length of session (mins) = 60	Intervention setting = care-home<br><br>Country = Ireland	Engagement type= participatory<br><br>Art type = music	Paired and unpaired t-tests (No test statistics reported); Significantly lower depression scores in int group compared with ctrl group ($p=.003$).

**Alves 2013**	Randomised<br><br>Control group type: Combined (usual activities and active)	Anxiety	Becks Anxiety Inventory	N = 65<br><br>Mean Age = 68.37 (7.36)<br><br>Sex = 92.31% F<br><br>Ethnicity/Race = no info	Non-Therapy	Length in weeks= 16<br><br>No. of sessions = 32<br><br>Length of session (mins) = 120	Intervention setting = community<br><br>Country = Brazil	Engagement type= participatory<br><br>Art type = dance	Group x Time Interaction: $F(2,62)=21.15$, $p<.001, \eta^2=.41$. Significantly lower anxiety scores in int group compared with usual activities control (p=.001) and active control (p=.005).
**Ayari et al., 2023**	Randomised<br><br>Control group type: Active	Depression	GDS-15	N = 23<br><br>Mean Age = 78 (range +/- 7 years)<br><br>Sex = 69.5% F<br><br>Ethnicity/Race = No info<br><br>MCI population	Non-Therapy	Length in weeks= 16<br><br>No. of sessions = 16<br><br>Length of session (mins) = 60	Intervention setting = Community<br><br>Country = France	Engagement type = participatory<br><br>Art type = dance	Repeated ANOVA (No test statistics reported); Time main effect ($\eta^2_p = 0.75$). Depression scores were significantly lower post-intervention for both the intervention (p<.001) and control groups (p<.001), but no significant difference between the groups.
**Berrol et al., 1997**	Non-Randomised<br><br>Control group type:	Depression*	Geriatric Depression Scale	N = 134<br><br>Mean Age = 74<br><br>Sex = 77% F	Therapy	Length in weeks= 21	Intervention setting = Mixed (care-home and	Engagement type= participatory	MANCOVA (No test statistics reported); No significant difference in depression scores

* Outcome results not included in meta-analysis

	usual activities			Ethnicity/Race = No info		No. of sessions = 42<br><br>Length of session (mins) = 45	community).<br><br>Country = USA	Art type = dance	between int and ctrl group (p=.435).
**Blumen et al. 2023**	Randomised<br><br>Control group type: Active	Depression*	Geriatric Depression Scale	N= 25<br><br>Mean Age = 76.45 (5.79)<br><br>Sex = 56% F<br><br>Ethnicity/Race (%) Native American 4; Black/African American 24; White 48; Mixed race 8; Unknown/not reported 16<br><br>MCI Population	Non-Therapy	Length in weeks= 24<br><br>No. of sessions = 48<br><br>Length of session (mins) = 90	Intervention Setting = Community<br><br>Country = USA	Engagement type = Participatory<br><br>Art Type = dance	Linear mixed effects models. No significant difference in depression scores between the intervention and control groups ($b=1.08$, $SE_b=1.11$; 95%CI -1.30-3.46).
**Chang et al. 2021**	Randomised<br><br>Control group type: usual activities	Depression	GDS – 15	N = 109<br><br>Mean Age = 76.29 (3.6)<br><br>Sex = 100% F	Non-Therapy	Length in weeks= 18<br><br>No. of sessions = 54	Intervention setting = care-home<br><br>Country = China	Engagement Type = participatory<br><br>Art type = dance	Group x Time effect. Significantly lower depression scores in int group compared with ctrl group ($B=.416$, $p<.001$;

				Ethnicity/Race: No info<br><br>MCI population		Length of sessions (mins) = 30			95%CI .228-.604; d=-.88)
**Ching-Teng et al. 2019**	Randomised<br><br>Control group type: usual activities	Depression	Geriatric Depression Scale (SF)	N = 55<br><br>Mean Age = 76.93 (8.33)<br><br>Sex = 62.1% F<br><br>Ethnicity/Race = no info	Non-Therapy	Length in weeks= 12<br><br>No. of sessions = 12<br><br>Length of session (mins) = 90 - 100	Intervention setting = care home<br><br>Country = Taiwan	Engagement type= participatory<br><br>Art type = visual art	Significant within group reduction in depression in int group (t=11.927, p<.001). No within group change in ctrl group (t=.466, p=.524). No dfs reported.
**Chippendale & Bear-Lehman. 2012**	Randomised<br><br>Control group type: waiting list	Depression	Geriatric Depression Scale (SF)	N = 45<br><br>Mean Age = 84.04 (7.56)<br><br>Sex = 68.9% F<br><br>Race = 75.6% white<br>11.1% black<br>4.4% Hispanic<br>9.9% Asian	Non-Therapy	Length in weeks= 8<br><br>No. of sessions = 8<br><br>Length of session (mins) = 90	Intervention setting = supported living<br><br>Country = USA	Engagement type= participatory<br><br>Art type = Creative writing	Significant Time x Group interaction with depression scores lower in int group compared with ctrl group (F(1,43)=5.10,p=.029)
**Ciasca et al. 2018**	Randomised<br><br>Control group type:	Depression<br><br>Anxiety	Geriatric Depression Scale	N = 56<br><br>Mean Age = 67.8 (6.2)	Therapy	Length in weeks= 20	Intervention setting = Community	Engagement type= participatory	Age-adjusted logistic regression (No test statistics reported). Significantly lower depression scores in

	usual activities		Becks Depression Inventory<br><br>Becks Anxiety Inventory	Sex = 100% F<br><br>Ethnicity/Race = N/A		No. of sessions = 20<br><br>Length of session (mins) = 90	Country = Brazil	Art type = Visual Art	int group for both GDS (p=.007) and BDI (p=.025) measures and anxiety scores (p=.032) in int group compared with ctrl group
**Cohen et.al. 2006**	Randomised<br><br>Control group type: usual activities	Depression	Geriatric Depression Scale (SF)	N = 166<br><br>Mean Age = 79.3<br><br>Sex = 79% F<br><br>Race = 92.5% white	Non-Therapy	Length in weeks= 30<br><br>No. of sessions = 30<br><br>Length of session (mins) = no info	Intervention setting = Community<br><br>Country = USA	Engagement type= participatory<br><br>Art type = music	ANCOVA (No test statistics reported). No significant differences between groups after controlling for pre-intervention depression score (No p-values reported).
**Coulton et al. 2015**	Randomised<br><br>Control group type: usual activities	Depression<br><br>Anxiety	HADS-D<br><br>HADS-A	N = 258<br><br>Mean Age = 69.2 (7.14)<br><br>Sex = 83.9% F<br><br>Race = 98% white	Non-Therapy	Length in weeks= 14<br><br>No. of sessions = No info<br><br>Length of session (mins) = 90	Intervention setting = community<br><br>Country = UK	Engagement type= participatory<br><br>Art type = music	ANCOVA (No test statistics reported). Significantly lower depression (p<.01) and anxiety (p<.01) int group compared with ctrl group at intervention end.<br><br>No significant differences between groups found at 6 months after intervention for

									depression (p=.14) or anxiety (p=.13).
**De Medeiros et al. 2009**	Randomised<br><br>Control group type: active	Depression	Geriatric Depression Scale (SF)	N =36<br><br>Mean Age = 80.7 (5.4)<br><br>Sex = 60.8% F<br><br>Ethnicity = 100% European Americans	Non-Therapy	Length in weeks= 8<br><br>No. of sessions = 8<br><br>Length of session (mins) = 90	Intervention setting = Supported Living<br><br>Country = USA	Engagement type= participatory<br><br>Art type = creative writing	Mixed ANOVA (No test statistics reported). No significant main effects or interactions between groups either post intervention or at 34 week follow-up.
**Dominguez et al. 2018**	Non-Randomised<br><br>Control group type: usual activities	Depression	Geriatric Depression Scale (SF)	N = 171<br><br>Mean Age = 69.1 (5.85)<br><br>Sex = 84.2% F<br><br>Ethnicity/Race = no info<br><br>MCI population	Non-Therapy	Length in weeks= 48<br><br>No. of sessions = 96<br><br>Length of session (mins) = 60	Intervention setting = community<br><br>Country = Philippines	Engagement type= participatory<br><br>Art type = dance	Independent t-test. Significantly lower depression in the int group compared to the ctrl group ($t(169)=-2.3$, $p=.02$; 95%CI=-1.5 to -0.1).
**Ellis-Hill et al. 2017**	Randomised<br><br>Control group type: usual activities	Depression<br><br>Anxiety	HADS -D<br><br>HADS-A	N = 47<br><br>Mean Age = 69.8 (12.13)<br><br>Sex = 43% F<br>Race<br>= 96% white	Non-Therapy	Length in weeks= 14<br><br>No. of sessions = 10<br><br>Length of session (mins) = 120	Intervention setting = community<br><br>Country = UK	Engagement type= participatory<br><br>Art type = visual art	ANCOVA used to estimate effect size (Cohen's d; No other test statistics reported). Effect size for depression: d=-.14 and anxiety: Cohen's d=-.45 in favour of int group.

				2% mixed white and Asian<br>2% black or black British African					
**Esmail et al. 2020**	Randomised<br><br>Control group type: waiting list	Anxiety[*]	STAI-State	N = 62<br><br>Mean Age = 67.48 (5.37)<br><br>Sex = 77.92% F<br><br>Ethnicity/Race = no info	Non-Therapy	Length in weeks= 12<br><br>No. of sessions = 36<br><br>Length of session (mins) = 60	Intervention setting = community<br><br>Country = Canada	Engagement type= participatory<br><br>Art type = dance	Mixed ANOVA. Group x Time interaction. Significant reduction in anxiety score in int group compared with ctrl (F (2,37)=5.01, p=.01)
**Eyigor et al. 2009**	Randomised<br><br>Control group type: usual activities	Depression[*]	Geriatric depression scale	N = 40<br><br>Mean Age = 72.4 (6.7)<br><br>Sex = 100% F<br><br>Ethnicity/Race = no info	Non-Therapy	Length in weeks= 8<br><br>No. of sessions = 24<br><br>Length of session (mins) = 60	Intervention setting = community<br><br>Country = Turkey	Engagement type= participatory<br><br>Art type = dance	Paired t-test (No test statistics reported). No significant difference between int and ctrl group (p>.05).
**Fausto et al. 2022**	Non-Randomised	Depression	Becks Depression Scale II	N= 64	Non-Therapy	Length in weeks = 20	Intervention setting = community	Engagement type = participatory	Repeated ANCOVA. Significant Group x Time interaction

[*] Outcome results not included in meta-analysis

	Control group type: Usual activities			Mean Age = 68.9 (6.7)<br><br>Sex = 88.7% F<br><br>Ethnicity = 100% African Americans		No. of sessions = 40<br><br>Length of session (mins) = 60	Country = USA	Art type = dance	(F(1,53)=7.42, p= .009) with depression scores reducing in the int group while remaining stable in the ctrl group.
**Galinha et al. 2021**	Randomised<br><br>Control group type: waiting list	Depression	DASS 21 Depression	N = 149<br><br>Mean Age = no info<br><br>Sex = no info<br><br>Ethnicity/Race = no info	Non-Therapy	Length in weeks = 16<br><br>No. of sessions =34<br><br>Session length (mins) = 120	Intervention setting = community<br><br>Country = Portugal	Engagement type = participatory<br><br>Art type = music	Mixed ANOVA. Group x Time interaction. No significant differences between groups for depression (F=0, p=.95) and anxiety (F=1.20, p=.276). No dfs reported.
**Gök Ugur et al. 2017**	Randomised<br><br>Control group type: usual activities	Depression	Geriatric Depression Scale (SF)	N = 64<br><br>Mean Age = 76.8 (7.8)<br><br>Sex = 34.4% F<br><br>Ethnicity/Race = no info	Therapy	Length in weeks= 8<br><br>No. of sessions = 24<br><br>Length of session (mins) = 50	Intervention setting = care-home<br><br>Country = Turkey	Engagement type= receptive<br><br>Art type = music	Independent t-test. Significantly lower depression scores in int group compared with ctrl group (t=-2.861, p=.006). No dfs reported.
**Ilali et al. 2018**	Randomised<br><br>Control group type: usual activities	Depression	Geriatric Depression Scale	N = 54<br><br>Mean Age = 70<br><br>Sex = 64.8 %	Non-Therapy	Length in weeks= 6<br><br>No. of sessions = 6	Intervention setting = community<br><br>Country = Iran	Engagement type= participatory<br><br>Art type =visual art	t-tests and Mixed ANOVA (t-statistics not reported). Significantly reduced depression in the int group compared to

				Ethnicity/Race = no info		Length of session (mins) = 60			the ctrl group both at one week post intervention (p<.001; 95%CI=1.098-3.098) and at one month post intervention (p<.001; 95%CI=2.531-4.653); F(2,156)=7.18, p=.001.
**Johnson et al. 2018**	Randomised<br><br>Control group type: waiting list control	Depression	PHQ-8	N = 390<br><br>Mean Age = 71.3 (7.2)<br><br>Sex = 76% F<br><br>Race = 35% non-Latino white, 26% Non-Latino black, 20% Asian, 18.5% Latino	Non-Therapy	Length in weeks= 44<br><br>No. of sessions = 44<br><br>Length of session (mins) = 90	Intervention setting = community<br><br>Country = USA	Engagement type = participatory<br><br>Art type = music	Linear mixed models. Group x Time effect. No significant difference between groups (β=-.19, p=.62)
**Kaasgaard et al. 2022**	Randomised<br><br>Control Group Type: Active	Depression<br><br>Anxiety	HADS<br><br>HADS	N=270<br><br>Mean Age = 69.55 (8.4)<br><br>Sex = 62.22 % F<br>Ethnicity/ Race = no info	Non-Therapy	Length in weeks= 10<br><br>No. of sessions = 20	Intervention setting = Community<br><br>Country = Denmark	Engagement Type = Participatory<br><br>Art Type = music	t-tests (No test statistics reported). No significant between group differences for either depression (p=.19; 95%CI=-.1 to .7) or anxiety (p=.33; 95%CI=-.9 to.3)

						Length of session (mins) = 90			
**Kim 2013**	Randomised<br><br>Control group type: usual activities	Anxiety	STAI 1 (state)	N = 50<br><br>Mean Age = 78.76 (4.02)<br>Sex = 78%<br><br>Ethnicity = 100% Korean American	Therapy	Length in weeks= 4<br><br>No. of sessions = 12<br><br>Length of session (mins) = 60 - 75	Intervention setting = community<br><br>Country = USA	Engagement type= participatory<br><br>Art type = visual art	Independent t-test. Significant decrease in anxiety in int group compared with ctrl (t(47)=-10.81, p=.001)
**Kim & Kang 2021**	Randomised<br><br>Control group type: Active	Depression*<br><br>Anxiety*	Geriatric Depression Scale SF<br><br>Geriatric Anxiety Index	N = 40<br><br>Mean Age = 81.6<br><br>Sex = 77.5% F<br><br>Ethnicity/Race = no info	Non-Therapy	Length in weeks =12<br><br>No. of sessions = 24<br><br>Length of session (mins) = 50	Intervention setting = care-home<br><br>Country = South Korea	Engagement Type = participatory<br><br>Art Type = music and movement	ANCOVA. Significantly lower depression (F=9.52, p<.001) and anxiety (F=18.21, p<.001) in int group compared to ctrl. dfs not reported.
**Lazarou et al., 2017**	Randomised<br><br>Control group type:	Depression	Geriatric Depression Scale	N = 129<br><br>Mean Age = 66.8 (10.1)	Non-Therapy	Length in weeks= 40	Intervention setting = community	Engagement type= participatory	Independent t-tests (No test statistics reported). Significantly lower

* Outcome results not included in meta-analysis

	usual activities			Sex = no info<br><br>Ethnicity/Race = no info<br><br>MCI population		No. of sessions = 80<br><br>Length of session (mins) = 60	Country = Greece	Art type = dance	depression in the int group compared with the ctrl group (p=.022).
**Lin et al. 2022**	Randomised<br><br>Control group type: Active control & Waitlist Control	Depression*<br><br>Anxiety*	GDS<br><br>SAS	N = 135<br><br>Mean Age = 70.93 (6.91)<br><br>Sex = 62.22% F<br><br>Ethnicity/Race = no info<br><br>MCI population	Non-Therapy	Length in weeks= 24<br><br>No. of sessions = 24<br><br>Length of session (mins) = 90	Intervention setting = community<br><br>Country = China	Engagement type = Participatory<br><br>Art Type = visual arts & storytelling	Linear mixed models (No test statistics reported). Depression scores fell significantly for participants in the intervention group compared with the control group post-intervention at 24 weeks (Int v. A-control: 95%CI=.699-5.104, p=.011; Int v. W-control: 95%CI=1.594-6.109, p=.001). No significant difference was found at the 48-week follow-up (No statistics reported).
**Liu et al. 2013**	Randomised<br><br>Control group type: no info	Depression	Hamilton depression scale	N = 50<br><br>Mean Age = no info<br><br>Sex = no info	Therapy	Length in weeks= 8<br><br>No. of sessions = 8	Intervention setting = care-home<br><br>Country = China	Engagement type= receptive<br><br>Art type = music	Independent t-test (No test statistics reported). Significant reduction in depression scores in int group compared

				Ethnicity/Race =no info		Length of session (mins) = 60-120			with ctrl group (p<.05). Significant difference maintained at 8 week follow-up (p<.05)
**Mahendran et al. 2018**	Randomised<br><br>Control group type: usual activities	Depression*<br><br>Anxiety*	Geriatric Depression Scale<br><br>Geriatric Anxiety Inventory	N = 68<br><br>Mean Age = 71.1 (5.3)<br><br>Sex = 82.4% F<br>Ethnicity/Race =no info<br><br>MCI population	Therapy	Length in weeks= 36<br><br>No. of sessions = 24<br><br>Length of session (mins) = 60	Intervention setting = Community<br><br>Country = Singapore	Engagement type= participatory<br><br>Art type = visual art & music	Linear mixed models (No test statistics reported). No significant difference between int and ctrl groups on depression at 3 months or at 6 months (all ps >=.431) or on anxiety at 3 months or at 6 months (all ps >=.444).
**Masika et al. 2021**	Randomised<br><br>Control group type: active	Depression	Geriatric Depression Scale (SF)	N = 39<br><br>Mean Age = 72.7 (7.1)<br><br>Sex = 87.2% F<br><br>Ethnicity/Race = no info	Therapy	Length in weeks= 6<br><br>No. of sessions = 12<br><br>Length of session	Intervention setting = community<br><br>Country = Tanzania	Engagement type= participatory<br><br>Art type = visual art	Generalised Estimating Equation (Group effect test statistic not reported). Significant reduction in depression in int group compared with ctrl group (Group

* Outcome results not included in meta-analysis

				MCI population		(mins) = no info			effect: p=.027) and between groups over time (Group × time interaction effect: β=−3.0, SE=.822], p<.001).
**Masika et al. 2022**	Randomised<br><br>Control group type: active	Depression	Geriatric Depression Scale	N = 127<br><br>Mean Age = 73.8 (8)<br><br>Sex = 78% F<br><br>Ethnicity/Race = no info	Therapy	Length in weeks = 6<br><br>No. of sessions = 12<br><br>Length of session (mins) = 120	Intervention setting = community<br><br>Country = Tanzania	Engagement type = participatory<br><br>Art type = visual art	Generalised Estimating Equation. Significantly reduced depression in int compared to control group (β =−2.01, 95% CI=−3.09−0.93, p<.001) post intervention. Significant differences not maintained at 3 month (β=−0.9, 95% CI:−2.1-0.3, p=.141) or 6 month (β=−1.3, 95% CI:−2.3-0.3, p=.116) follow-up.
**Mastel-Smith et al. 2007**	Randomised<br><br>Control group type: waiting list	Depression	Brief symptom inventory (Depression Subscale)	N = 33<br><br>Mean Age = 71.4 (8.8)<br><br>Sex = 81% F	Non-Therapy	Length in weeks= 10<br><br>No. of sessions = 10	Intervention setting = community<br><br>Country = USA	Engagement type= participatory<br><br>Art type = dance	Independent t-test. Significantly lower depression scores in int group compared to ctrl group (t (19.26)=–2.277, p=.036).

				Race = 94% white		Length of session (mins) = 120			
**Matto et al. 2015**	Randomised<br><br>Control group type: active	Depression	Geriatric Depression Scale (SF)	N = 19<br><br>Mean Age = no info<br><br>Sex = 45% F<br><br>Ethnicity/Race = no info	Non-Therapy	Length in weeks= 10<br><br>No. of sessions = no info<br><br>Length of session (mins) = 50-60	Intervention setting = care-home<br><br>Country = USA	Engagement type= participatory<br><br>Art type = music, imagery, and movement	Mixed ANOVA. Significantly lower depression scores in int group compared to ctrl group (F=4.534, p<.05). No dfs reported. At 6 week follow up scores regressed slightly back to pre-treatment scores with int group showing less regression than ctrl group.
**Mohammadi et al. 2011**	Randomised<br><br>Control group type: no info	Depression*<br><br>Anxiety	DASS-21 Depression<br><br>DASS-21 Anxiety	N = 19<br><br>Mean Age = 69.47<br><br>Sex = 47.37% F<br><br>Ethnicity/Race = no info	Therapy	Length in weeks= 10<br><br>No. of sessions = 10<br><br>Length of session (mins) = 90	Intervention setting = care-home<br><br>Country = Iran	Engagement type= participatory<br><br>Art type = music	Mann-Whitney U. Significant decrease in depression (U=.50, p<.001) and anxiety (U=9.00, p=.004) in int group compared to ctrl.

* Outcome results not included in meta-analysis

**Moore et al. 2017**	Randomised<br><br>Control group type: active	Depression<br><br>Anxiety	Geriatric Depression Scale<br><br>Becks Anxiety Inventory	N = 13<br><br>Mean Age = 76.7 (6.4)<br><br>Sex = 76.92% F<br><br>Race = 92.3 white	Non-Therapy	Length in weeks= 6<br><br>No. of sessions = 6<br><br>Length of session (mins) = 90	Intervention setting = community<br><br>Country = USA	Engagement type= participatory<br><br>Art type = drama	Mixed ANOVA. No significant difference between groups for depression (F=.53, p=.48, d=.03) or anxiety (F=.06, p=.82, d=.04). No dfs reported.
**Roswiyani et al. 2020**	Randomised<br><br>Control group type: usual activities	Depression	Becks Depression Index	N = 267<br><br>Mean Age = 73.82 (9.61)<br><br>Sex = 68.50% F<br><br>Ethnicity = Javanese 50.2%, Sumatra 10.1% Sulawesi 3.4% Kalimantan1.1 % Madura 33% Maluku 2.2%	Non-Therapy	Length in weeks= 8<br><br>No. of sessions = 16<br><br>Length of session (mins) = 90	Intervention setting = care home<br><br>Country = Indonesia	Engagement type= participatory<br><br>Art type = visual art	MANOVA. Significantly lower depression in both int groups (art group, art & Qiong group) compared with ctrl group (F(3, 263)= 3.523, p=.016; 95%CI=−3.20 to −1.19).
**Sangvanich et al., 2023**	Randomised<br><br>Control group type: Active	Depression	Thai GDS	N= 30<br><br>Mean Age = 71.40 (6.28)	Non-Therapy	Length in weeks= 6<br><br>No. of sessions = 6	Intervention setting = Community	Engagement Type = Participatory	No test statistics reported. No significant difference was found in depression scores

				Sex = 83.33<br><br>Ethnicity/Race = No info<br><br>MCI population		Length of session (mins) = 60	Country = Thailand	Art Type = visual arts	between the intervention and control group. Sad mood scores of both the int and ctrl groups were lower but not significantly different after the intervention (Art: p=.304, Control: p=.769)
**Supiano et al. 1989**	Non-Randomised<br><br>Control group type: usual activities	Depression*	Geriatric Depression Scale	N = 116<br><br>Mean Age = 83.4<br><br>Sex = 75% F<br><br>Ethnicity/Race = no info	Non-Therapy	Length in weeks= 8<br><br>No. of sessions = 8<br><br>Length of session (mins) = 60	Intervention setting = care-home<br><br>Country = USA	Engagement type= participatory<br><br>Art type = creative writing	Paired t-tests (No test statistics reported). Some decline in depression scores in int group approaching significance (p<.08).
**Vankova et al. 2014**	Randomised<br><br>Control group type: usual activities	Depression	Geriatric Depression Scale (SF)	N = 162<br><br>Mean Age = 83.11 (7.98)<br><br>Sex = 92% F<br><br>Ethnicity/Race = no info	Non-Therapy	Length in weeks= 12<br><br>No. of sessions = 12<br><br>Length of session (mins) = 60	Intervention setting = care-home<br><br>Country = Czech Republic	Engagement type= participatory<br><br>Art type = dance	Paired t-tests (No test statistics reported). Significantly lower depression scores within int group (p=.005) compared to within ctrl group (p=.081). 2x2 GLM group: ctrl vs int; test: pretest vs post-

*Outcome results not included in meta-analysis

									test (Wilks lambda F=10.97, p=.001).
**Vrinceanu et al.2019**	Randomised<br><br>Control group type: waiting list	Depression	Geriatric Depression Scale	N = 62<br><br>Mean Age = 67.45 (5.32)<br><br>Sex = 75%<br><br>Ethnicity/Race = no info	Non-Therapy	Length in weeks= 12<br><br>No. of sessions = 36<br><br>Length of session (mins) = 60	Intervention setting = community<br><br>Country = Canada	Engagement type= participatory<br><br>Art type = dance	Mixed ANOVA. No significant differences between the int group and the active ctrl or the usual activities ctrl (Group x Time Interaction: F=.56, p=.58). No dfs reported.
**Wang et al. 2020**	Non-Randomised<br><br>Control group type: usual activities	Depression	Geriatric Depression Scale	N = 66<br><br>Mean Age = 81.08 (6.36)<br><br>Sex = 71.21% F<br><br>Ethnicity/Race = no info<br><br>MCI population	Non-Therapy	Length in weeks= 12<br><br>No. of sessions = 36<br><br>Length of session (mins) = 40	Intervention setting = care-home<br><br>Country = China	Engagement type= participatory<br><br>Art type = dance	Linear mixed effects model (No test statistics reported). Significantly lower depression scores in the int group compared with the control group (p<.001).
**Wang et al., 2023**	Randomised<br><br>Control group type: Usual Activities	Depression	GDS-15	N=38<br><br>Mean Age = 71.95 (6.7)<br><br>Sex= 71.05% F	Non-Therapy	Length in weeks= 6<br><br>No. of sessions = 12	Intervention Setting = Community<br><br>Country = Taiwan	Engagement type = Participatory<br><br>Art Type = magic & visual arts	Repeated ANOVA. Group x Time interaction: F(1, 36)=7.35, p=.010. Post intervention depression scores were significantly

				Ethnicity/Race = no info		Length of session (mins) = 90			lower than pre-intervention scores for the intervention group (p=.004).
**Watson et al. 2023**	Randomised<br><br>Control group type: Usual Activities	Depression*	GDS-15	N=252<br><br>Mean Age = 71.44 (5.29)<br><br>Sex= 82% F<br><br>Ethnicity/Race = no info	Non-Therapy	Length in weeks= 64<br><br>No. of sessions = 64<br><br>Length of session (mins) = 120	Intervention Setting = community<br><br>Country = Canada	Engagement type = Participatory<br><br>Art Type = Visual Arts & Drama	Ordinary Least Squares regression. A trend in the time x treatment interaction for the arts intervention group and depression (b=-1.263, SE_b=.770, p=0.10) with depression approaching reduction over time. This reduction was most notable in participants with higher pre-intervention depression scores.
**Xue et al., 2023**	Randomised<br><br>Control group type: Usual Activities	Depression	GDS-15	N=80<br><br>Mean Age = 75.43 (4.75)<br><br>Sex = 77.50% F<br><br>Ethnicity/Race = no info	Therapy	Length in weeks= 8<br><br>No. of sessions = 32<br><br>Length of session (mins) = no info	Intervention Setting = care-home<br><br>Country = China	Engagement type = non-participatory<br><br>Art Type = music	Multiple linear regression. Post-intervention depression scores were significantly lower for the intervention group compared with the control group (95%CI=−3.28 to −1.79, p<.001).

**Yan et al. 2020**	Non-Randomised<br><br>Control group type: waiting list	Depression*<br><br>Anxiety	Geriatric Depression Scale<br><br>Zung Self Rating Anxiety Scale	N= 48<br><br>Median Age = 68<br><br>Sex = 60.4% F<br><br>Ethnicity/Race = No info<br><br>MCI population	Non-Therapy	Length in weeks = 6<br><br>No. of sessions = 12<br><br>Length of session (mins) = 60-90	Intervention setting = Community<br><br>Country = China	Engagement type = participatory<br><br>Art Type = visual art	Independent samples t-test. Significant reduction in depression (t=-2.15, p=.037) and anxiety (t=-3.15, p=.003) scores in int group compared with ctrl
**Yap et al. 2017**	Randomised<br><br>Control group type: usual activities	Depression*	Geriatric Depression Scale	N = 54<br><br>Mean Age = 74.65 (6.4)<br><br>Sex = 94% F<br><br>Ethnicity = 100% Chinese	Non-Therapy	Length in weeks= 11<br><br>No. of sessions = 10<br><br>Length of session (mins) = 60	Intervention setting = community<br><br>Country = Singapore	Engagement type= participatory<br><br>Art type = music	Non-significant difference in depression scores between groups either post intervention (11 weeks) or at follow-up (22 weeks; Coefficient=-.479, p=.245; 95%CI= -1.287 to 0.329)
**Yu et al., 2022**	Randomised<br><br>Control Group Type: Usual Activities	Depression	GDS-15	N = 63<br><br>Mean Age = 79.68 (7.27)<br><br>Sex = 71.42% F	Non-Therapy	Length in weeks= 10<br><br>No. of sessions = 20	Intervention Setting = Care-home<br><br>Country = Taiwan	Engagement Type = non-participatory<br><br>Art Type = music	Generalised Estimating Equation. Post-intervention depression scores were significantly lower for the

*Outcome results not included in meta-analysis

				Ethnicity/Race = no info		Length of session (mins) = 30			intervention group compared with the control group (Group x Time effect: Wald $\chi^2(1)$=-.78,p<.0001)
**Zhang et al. 2023**	Randomised<br><br>Control Group Type: Usual Activities	Depression	GDS-15	N = 72<br><br>Mean Age = 64.75 (4.35)<br><br>Sex = 63.88% F<br><br>Ethnicity/Race = no info	Non-Therapy	Length in Weeks = 16<br><br>No. of sessions = 80<br><br>Length of session = 60	Intervention setting= Community<br><br>Country = China	Engagement type = Participatory<br><br>Art type = dance	Mixed ANOVA. Post-intervention depression scores were significantly lower for the intervention group compared with the control group (Group x Time interaction: F= 29.634, p<.001). No dfs reported.
**Zhao et al. 2021**	Non-Randomised<br><br>Control group type: usual activities	Depression	Geriatric Depression Scale	N= 63<br><br>Mean age = 72.29 (6.03)<br><br>Sex = 82.5 % F<br><br>Ethnicity/Race = No information<br><br>MCI population	Non-Therapy	Length in weeks = 12<br><br>No. of sessions = 36<br><br>Length of session (mins) = 60	Intervention setting = community<br><br>Country = China	Engagement type = participatory<br><br>Art type = dance	Linear mixed effects modelling. Significantly lower depression scores in the int group compared with the control group both post intervention (12 weeks; p<.001) and at 3-month post intervention follow-up (24 weeks; p<.001). Group x Time effect: F= 25.73, p<.001).

**Zhu et al. 2018**	Randomised<br><br>Control group type: usual activities	Depression	Geriatric Depression Scale (SF)	N = 60<br><br>Mean Age = 69.6 (7)<br><br>Sex = 60% F<br><br>Ethnicity = no info<br><br>MCI population	Non-Therapy	Length in weeks= 12<br><br>No. of sessions = 36<br><br>Length of session (mins) = 35	Intervention setting = community<br><br>Country = China	Engagement type= participatory<br><br>Art type = dance	Linear mixed effects modelling (No test statistics reported). No significant difference between groups for depression. Either immediately post intervention (95%CI=-1.3 to 4.1) or at follow-up (95%CI=-1.9 to 4.2). The control group had significant within group reduction of depression scores (95%CI=-5.2 to -1.5).

ANCOVA=Analysis of covariance; ANOVA=Analysis of variance; b=unstandardized beta; β=standardized beta; CI=confidence interval; d=Cohen's d (effect size); df=Degrees of freedom; F=F-statistic; GLM=General Linear Model; p=p-value (where p<.05 is statistically significant); SE=standard error; t=t-statistic

Appendix B

References Included in the meta-analysis

Adam, D., Ramli, A., & Shahar, S. (2016). Effectiveness of a combined dance and relaxation intervention on reducing anxiety and depression and improving quality of life among the cognitively impaired elderly. *Sultan Qaboos University Medical Journal*, *16*(1), e47.

Ahessy, B. (2016). The use of a music therapy choir to reduce depression and improve quality of life in older adults–A randomized control trial. *Music and Medicine*, *8*(1), 17-28.

Alves, H. (2013). *Dancing and the aging brain: The effects of a 4-month ballroom dance intervention on the cognition of healthy older adults* (Doctoral dissertation, University of Illinois at Urbana-Champaign). Retrieved online: https://core.ac.uk/download/pdf/17354697.pdf

Ayari, S., Abellard, A., Sakrani, S., Krinitskaia, A., Grzelak, M., Nader, R. B., & Gavarry, O. (2023). Comparison of dance and aerobic exercise on cognition and neuropsychiatric symptoms in sedentary older adults with cognitive impairment. *European Geriatric Medicine*, *14*(6), 1289-1299.

Berrol, C. F. (1997). Dance/movement therapy with older adults who have sustained neurological insult: A demonstration project. *American Journal of Dance Therapy*, *19*(2), 135-160.

Blumen, H. M., Ayers, E., Wang, C., Ambrose, A. F., Jayakody, O., & Verghese, J. (2022). Randomized controlled trial of social ballroom dancing and treadmill walking: preliminary findings on executive function and neuroplasticity from dementia-at-risk older adults. *Journal of aging and physical activity*, *31*(4), 589-599.

Chang, J., Zhu, W., Zhang, J., Yong, L., Yang, M., Wang, J., & Yan, J. (2021). The effect of Chinese square dance exercise on cognitive function in older women with mild cognitive impairment: the mediating effect of mood status and quality of life. *Frontiers in Psychiatry*, *12*; doi: 10.3389/fpsyt.2021.711079

Ching-Teng, Y., Ya-Ping, Y., & Yu-Chia, C. (2019). Positive effects of art therapy on depression and self-esteem of older adults in nursing homes. *Social Work in Health Care*, *58*(3), 324-338.

Chippendale, T., & Bear-Lehman, J. (2012). Effect of life review writing on depressive symptoms in older adults: A randomized controlled trial. *The American Journal of Occupational Therapy*, *66*(4), 438-446.

Ciasca, E. C., Ferreira, R. C., Santana, C. L., Forlenza, O. V., Dos Santos, G. D., Brum, P. S., & Nunes, P. V. (2018). Art therapy as an adjuvant treatment for depression in elderly women: a randomized controlled trial. *Brazilian Journal of Psychiatry*, *40*, 256-263.

Cohen, G. D., Perlstein, S., Chapline, J., Kelly, J., Firth, K. M., & Simmens, S. (2006). The impact of professionally conducted cultural programs on the physical health, mental health, and social functioning of older adults. *The Gerontologist*, *46*(6), 726-734.

Coulton, S., Clift, S., Skingley, A., & Rodriguez, J. (2015). Effectiveness and cost-effectiveness of community singing on mental health-related quality of life of older people: randomised controlled trial. *The British Journal of Psychiatry*, *207*(3), 250-255.

De Medeiros, K., Mosby, A., Hanley, K. B., Pedraza, M. S., & Brandt, J. (2011). A randomized clinical trial of a writing workshop intervention to improve autobiographical memory and well-being in older adults. *International Journal of Geriatric Psychiatry*, *26*(8), 803-811.

Dominguez, J. C., Del Moral, M. C. O., Chio, J. O. A., de Guzman, M., Fe, P., Natividad, B. P., Decena, J. M., Montalvo, M. J. Y., Reandelar, M., & Phung, K. T. (2018). Improving cognition through dance in older filipinos with mild cognitive impairment. *Current Alzheimer Research*, *15*(12), 1136-1141.

Ellis-Hill, C., Thomas, S., Gracey, F., Lamont-Robinson, C., Cant, R., Marques, E. M., ... & Jenkinson, D. (2019). HeART of Stroke: randomised controlled, parallel-arm, feasibility study of a community-based arts and health intervention plus usual care compared with usual care to increase psychological well-being in people following a stroke. *BMJ Open*, *9*(3), e021098. doi: 10.1136/bmjopen-2017-021098

Esmail, A., Vrinceanu, T., Lussier, M., Predovan, D., Berryman, N., Houle, J., Karelis, A., Grenier, S., Minh Vu, T. T., Villalpando, J. M., & Bherer, L. (2020). Effects of dance/movement training vs. aerobic exercise training on cognition, physical fitness and quality of life in older adults: a randomized controlled trial. *Journal of Bodywork and Movement Therapies*, *24*(1), 212-220.

Eyigor, S., Karapolat, H., Durmaz, B., Ibisoglu, U., & Cakir, S. (2009). A randomized controlled trial of Turkish folklore dance on the physical performance, balance, depression and quality of life in older women. *Archives of Gerontology and Geriatrics*, *48*(1), 84-88.

Fausto, B. A., Azimipour, S., Charles, L., Yarborough, C., Grullon, K., Hokett, E., Duberstein, P. R., & Gluck, M. A. (2022). Cardio-dance exercise to improve cognition and mood in older African Americans: a propensity-matched cohort study. *Journal of Applied Gerontology*, *41*(2), 496-505.

Galinha, I. C., Fernandes, H. M., Lima, M. L., & Palmeira, A. L. (2021). Intervention and mediation effects of a community-based singing group on older adults' perceived physical and mental health: The Sing4Health randomized controlled trial. *Psychology & Health*, *38*, 1-21.

Gök Ugur, H., Yaman Aktaş, Y., Orak, O. S., Saglambilen, O., & Aydin Avci, İ. (2017). The effect of music therapy on depression and physiological parameters in elderly people living in a Turkish nursing home: a randomized-controlled trial. *Aging & Mental Health*, *21*(12), 1280-1286.

Ilali, E. S., Mokhtary, F., Mousavinasab, N., & Tirgari, A. H. (2018). Impact of art-based life review on depression symptoms among older adults. *Art Therapy*, *35*(3), 148-155.

Johnson, J., Stewart, A., Anna, N., Acree, M., Flatt, J., Max, W., & Gregorich, S. (2018). A choir intervention to promote well-being among diverse older adults: the community of voices trial. *Innovation in Aging*, *2*(Suppl 1), 455. https://doi.org/10.1093/geroni/igy023.1705

Kaasgaard, M., Rasmussen, D. B., Andreasson, K. H., Hilberg, O., Løkke, A., Vuust, P., & Bodtger, U. (2022). Use of Singing for Lung Health as an alternative training modality within pulmonary rehabilitation for COPD: a randomised controlled trial. *European Respiratory Journal*, *59*(5).

Kim, S. K. (2013). A randomized, controlled study of the effects of art therapy on older Korean-Americans' healthy aging. *The Arts in Psychotherapy*, *40*(1), 158-164.

Kim, H. S., & Kang, J. S. (2021). Effect of a group music intervention on cognitive function and mental health outcomes among nursing home residents: A randomized controlled pilot study. *Geriatric Nursing*, *42*(3), 650-656.

Lazarou, I., Parastatidis, T., Tsolaki, A., Gkioka, M., Karakostas, A., Douka, S., & Tsolaki, M. (2017). International ballroom dancing against neurodegeneration: a randomized controlled trial in Greek community-dwelling elders with mild cognitive impairment. *American Journal of Alzheimer's Disease & Other Dementias®*, *32*(8), 489-499.

Lin, R., Luo, Y. T., Yan, Y. J., Huang, C. S., Chen, L. L., Chen, M. F., Lin, M-J & Li, H. (2022). Effects of an art-based intervention in older adults with mild cognitive impairment: a randomised controlled trial. *Age and Ageing*, *51*(7), afac144.

Liu, X., Niu, X., Feng, Q., & Liu, Y. (2014). Effects of five-element music therapy on elderly people with seasonal affective disorder in a Chinese nursing home. *Journal of Traditional Chinese Medicine*, *34*(2), 159-161.

Mahendran, R., Gandhi, M., Moorakonda, R. B., Wong, J., Kanchi, M. M., Fam, J., Rawtaer, I., Kumar, A. P., Feng, L., & Kua, E. H. (2018). Art therapy is associated with sustained improvement in cognitive function in the elderly with mild neurocognitive disorder: Findings from a pilot randomized controlled trial for art therapy and music reminiscence activity versus usual care. *Trials*, *19*(1), 1-10.

Masika, G. M., Yu, D. S., & Li, P. W. (2021). Can visual art therapy be implemented with illiterate older adults with mild cognitive impairment? A pilot mixed-method randomized controlled trial. *Journal of Geriatric Psychiatry and Neurology*, *34*(1), 76-86.

Masika, G. M., Yu, D. S., Li, P. W., Lee, D. T., & Nyundo, A. (2022). Visual art therapy and cognition:

Effects on people with mild cognitive impairment and low education level. *The Journals of Gerontology: Series B*, *77*(6), 1051-1062.

Mastel-Smith, B. A., McFarlane, J., Sierpina, M., Malecha, A., & Haile, B. (2007). Improving depressive symptoms in community-dwelling older adults: A psychosocial intervention using life review and writing. *Journal of Gerontological Nursing*, *33*(5), 13-19.

Matto, H. C., Tompkins, C. J., Ihara, E. S., Inoue, M., & Byrd, A. (2015). Results from a music, imagery, and movement treatment intervention in a long-term care facility. *Families in Society*, *96*(4), 277-283.

Mohammadi, A. Z., Shahabi, T., & Panah, F. M. (2011). An evaluation of the effect of group music therapy on stress, anxiety and depression levels in nursing home residents. *Canadian Journal of Music Therapy*, *17*(1), 55-68.

Moore, R. C., Straus, E., Dev, S. I., Parish, S. M., Sueko, S., & Eyler, L. T. (2017). Development and pilot randomized control trial of a drama program to enhance well-being among older adults. *The Arts in Psychotherapy*, *52*, 1-9.

Roswiyani, R., Hiew, C. H., Witteman, C. L., Satiadarma, M. P., & Spijker, J. (2020). Art activities and qigong exercise for the well-being of older adults in nursing homes in Indonesia: a randomized controlled trial. *Aging & Mental Health*, *24*(10), 1569-1578.

Sangvanich, K., Tawankanjanachot, I., & Tangwongchai, S. (2023). Arts-Based Interventions to Enhance the Quality of Life and Cognition of the Elderly with Mild Cognitive Impairment. *Journal of Urban Culture Research*, *26*, 280-297.

Supiano, K. P., Ozminkowski, R. J., Campbell, R., & Lapidos, C. (1989). Effectiveness of writing groups in nursing homes. *Journal of Applied Gerontology*, *8*(3), 382-400.

Vankova, H., Holmerova, I., Machacova, K., Volicer, L., Veleta, P., & Celko, A. M. (2014). The effect of dance on depressive symptoms in nursing home residents. *Journal of the American Medical Directors Association*, *15*(8), 582-587.

Vrinceanu, T., Esmail, A., Berryman, N., Predovan, D., Vu, T. T. M., Villalpando, J. M., Pruessner, J. C., & Bherer, L. (2019). Dance your stress away: Comparing the effect of dance/movement training to aerobic exercise training on the cortisol awakening response in healthy older adults. *Stress*, *22*(6), 687-695.

Wang, S., Yin, H., Meng, X., Shang, B., Meng, Q., Zheng, L., Wang, L., & Chen, L. (2020). Effects of Chinese square dancing on older adults with mild cognitive impairment. *Geriatric Nursing*, *41*(3), 290-296.

Wang, W. L., Lee, K. T., Lin, W. C., Yang, Y. C., & Tsai, C. L. (2023). The effects of a magic-based intervention on self-esteem, depressive symptoms, and quality of life among community-dwelling older adults: a randomised controlled trial. *Psychogeriatrics*, *23*(4), 701-712.

Watson, B., Das, A., Maguire, S., Fleet, G., & Punamiya, A. (2024). The little intervention that could: creative aging implies healthy aging among Canadian seniors. *Aging & Mental Health*, *28*(2), 307-318.

Xue, B., Meng, X., Liu, Q., & Luo, X. (2023). The effect of receptive music therapy on older adults with mild cognitive impairment and depression: a randomized controlled trial. *Scientific Reports*, *13*(1), 22159.

Yan, Y. J., Lin, R., Zhou, Y., Luo, Y. T., Cai, Z. Z., Zhu, K. Y., & Li, H. (2021). Effects of expressive arts therapy in older adults with mild cognitive impairment: A pilot study. *Geriatric Nursing*, *42*(1), 129-136.

Yap, A. F., Kwan, Y. H., Tan, C. S., Ibrahim, S., & Ang, S. B. (2017). Rhythm-centred music making in community living elderly: a randomized pilot study. *BMC Complementary and Alternative Medicine*, *17*(1), 1-8.

Yu, A. L., Lo, S. F., Chen, P. Y., & Lu, S. F. (2022). Effects of group music intervention on depression for elderly people in nursing homes. *International Journal of Environmental Research and Public Health*, *19*(15), 9291.

Zhang, X., van der Schans, C. P., Liu, Y., Krijnen, W. P., & Hobbelen, J. S. M. (2023). Efficacy of dance intervention for improving frailty among chinese older adults living in the community: A randomized controlled trial. *Journal of Aging and Physical Activity*, *31*(5), 806-814.

Zhao, Y., Yin, M., Yao, X., & Li, Z. (2021). Effects of nurse-led square dancing on older patients with mild cognitive impairment combined with depressive symptoms: A pilot study. *Geriatric Nursing*, *42*(5), 1164-1171.

Zhu, Y., Wu, H., Qi, M., Wang, S., Zhang, Q., Zhou, L., Wang, S., Wang, W., Wu, T., Xiao, M., Yang, S., Chen, H., Zhang, L., Zhang, K., C., Ma, J., & Wang, T. (2018). Effects of a specially designed aerobic dance routine on mild cognitive impairment. *Clinical Interventions In Aging*, *13*, 1691.

Appendix C

Funnel Plot of the effect of group arts interventions on depression with imputed studies

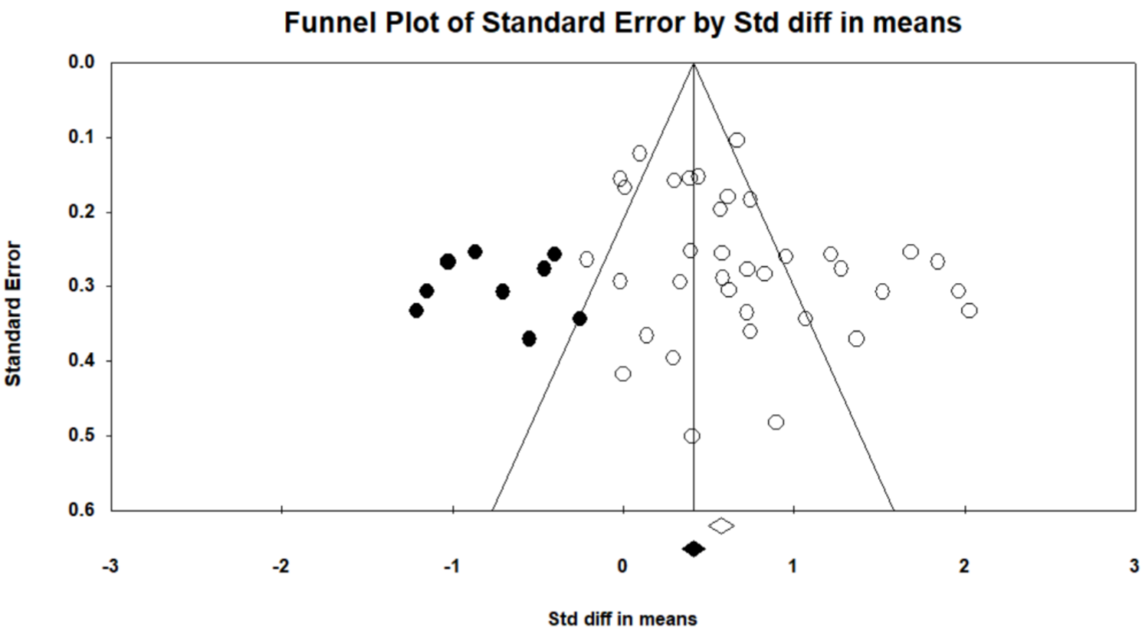

Appendix D

Subgroup Analyses for Depression

Table 2. Subgroup analysis for categorical moderators of group arts interventions on depression

	Subgroup	Study N	SMD	95% CI	z Value	p Value	I^2(%)	BGE
**All studies**	-	36	.70	.52-.87	7.83	.000	80.57	-
**Art type**	Visual Art	8	.77	.43-1.10	4.49	.000	71.08	.736
	Dance	13	.64	.38-.91	4.71	.000	76.14	
	Music	10	.81	.41-1.21	4.01	.000	90.77	
	Writing	2	-	-	-	-	-	
	Drama	1	-	-	-	-	-	
	Mix	2	-	-	-	-	-	
**Nature of Intervention**	Non -Therapy	29	.63	.44-.83	6.46	.000	81.03	.102
	Therapy	7	.97	.62-1.32	5.38	.000	66.79	
**Study Setting**	Community	22	.51	.32-.70	5.32	.000	73.21	.005
	Care home	12	1.07	.72-1.42	6.04	.000	84.24	
	Senior retirement community	2	-	-	-	-	-	
**Active/Receptive Engagement**	Active	32	.63	.46-.80	7.28	.000	77.08	.120
	Receptive	4	1.24	.49-1.98	3.25	.000	86.64	
**Country SES**	High income	20	.61	.37-.85	5.02	.000	81.34	.301
	Low-Middle income	16	.80	.55-1.04	6.34	.000	77.22	
**Control Type**	Active	7	.71	.20-1.22	2.74	.000	84.66	.207
	Usual Activity	21	.83	.60-1.07	6.88	.000	80.54	
	Waiting List	5	.45	.11-.80	2.56	.010	66.68	
	Mixed	1	-	-	-	-	-	
	No Info	2	-	-	-	-	-	
**Average Depression Score**	Depression	23	.81	.56-1.06	6.40	.000	82.34	.063
	No Depression	13	.49	.27-.71	4.35	.000	71.39	

SMD = standardised mean difference; CI = Confidence Interval; I^2= heterogeneity statistic; BGE = between group effect; All analyses were two-tailed. Adjusting for multiple comparisons using a Bonferroni Correction (alpha/number of tests, here .05/7) suggests that the any p-value less than .008 is statistically significant.

Appendix E

Meta-Regression Analyses for Depression

Table 3. Meta-regression analysis for continuous moderators of group arts interventions on depression

Depression Meta-Regression Results Table

Covariate	Study n	b	SE_b	Lower 95% CI	Higher 95% CI	z Value	*p* Value
**Average Age**	33	.01	.02	-.03	.04	.25	.800
**Intervention length (weeks)**	36	-.01	.01	-.02	.01	-1.22	.222
**Length of Sessions (mins)**	34	-.001	.004	-.008	.007	-0.25	.805
**Number of sessions**	33	-.003	.003	-.009	.004	-0.93	.354
**Intervention intensity (number of minutes per week)**	31	-.0004	.001	-.003	.003	-.24	.808

b = Unstandardised beta coefficient; SE_b = Standard error of b; CI = Confidence Interval

Appendix F

Subgroup Analyses for Anxiety

Table 4. Subgroup analysis for categorical moderators of group arts interventions on anxiety

	Subgroup	Study n	SMD	95% CI	z value	p value	I^2(%)	BGE
**All studies**		10	0.76					
**Nature of Intervention**	Non -Therapy	7	0.68	.20-1.15	2.76	.006	88.58	.459
	Therapy	3	0.98	.34-1.62	2.99	.003	61.61	
**Country SES**	High income	5	0.53	.07-1.12	1.12	.05	90.131	.169
	Mid/low income	5	1.00	.67-1.34	5.86	.000	38.42	
**Average Anxiety Score**	Anxiety	5	0.92	.49-1.35	4.19	.000	62.13	.436
	No Anxiety	5	0.63	.06-1.21	2.15	.032	89.65	

SMD = standardised mean difference; CI = Confidence Interval; I^2= heterogeneity statistic; BGE = between group effect; All analyses were two-tailed; Adjusting for multiple comparisons using a Bonferroni Correction (alpha/number of tests, here .05/3) suggests that the any p-value less than .017 is statistically significant.

Appendix G

Meta-Regression Analyses for Anxiety

Table 5. Meta-regression analysis for continuous moderators of group arts interventions on anxiety

Covariate	Study n	b	SE_b	Lower 95% CI	Higher 95% CI	z-value	p value
**Average Age**	9	.030	.061	-.085	.155	.41	.684
**Intervention length (weeks)**	10	-.037	.042	-.012	-.046	-.88	.378
**Length of Sessions (hours)**	10	-.014	.012	-.034	.007	-2.48	.186
**Number of Sessions**	9	.007	.032	-.069	.057	-.21	.837
**Intervention intensity (number of minutes per week)**	9	.002	.005	-.006	.011	.51	.612

b = Unstandardised beta coefficient; SE_b = Standard error of b; CI = Confidence Interval

Appendix H

Search Strategy

This appendix details the search strategy used. Please note that only depression and anxiety outcomes are presented in this paper.

Population: older adult* OR over 55 OR over 65 OR senior* OR older population OR elderly OR geriatric*.

Intervention Delivery: social OR group OR club

Intervention Type: art* OR artistic OR creative OR dance OR music OR singing OR song-writing OR drumming OR theatre OR performing arts OR acting OR visual arts OR drawing OR painting OR pottery OR sculpture OR cultural OR museum* OR festival OR gallery* OR drama* OR writing* OR autobiographical OR film Or art therapy* OR music therapy Or dance movement therapy OR dramatherapy OR poetry OR creative writing

Comparison: Randomised controlled Trial OR RCT OR quasi experimental OR pre-post OR baseline AND/OR evaluation.

Outcome: wellbeing OR mood OR mental health OR depression OR anxiety OR quality of life OR life satisfaction OR cognitive OR cognitive function* OR MOCA OR MMSE OR Health OR Physical

Additional Outcome: social isolation, loneliness, social connection

Setting: community OR neighbourhood OR care home OR nursing home OR retirement home OR assisted living Or sheltered living

Appendix I

Risk of Bias

This appendix presents the risk of bias plots created using the Robvis tool (McGuinness & Higgins, 2021)

Figure 1. Summary Plot for Risk of Bias in Non-Controlled Trials

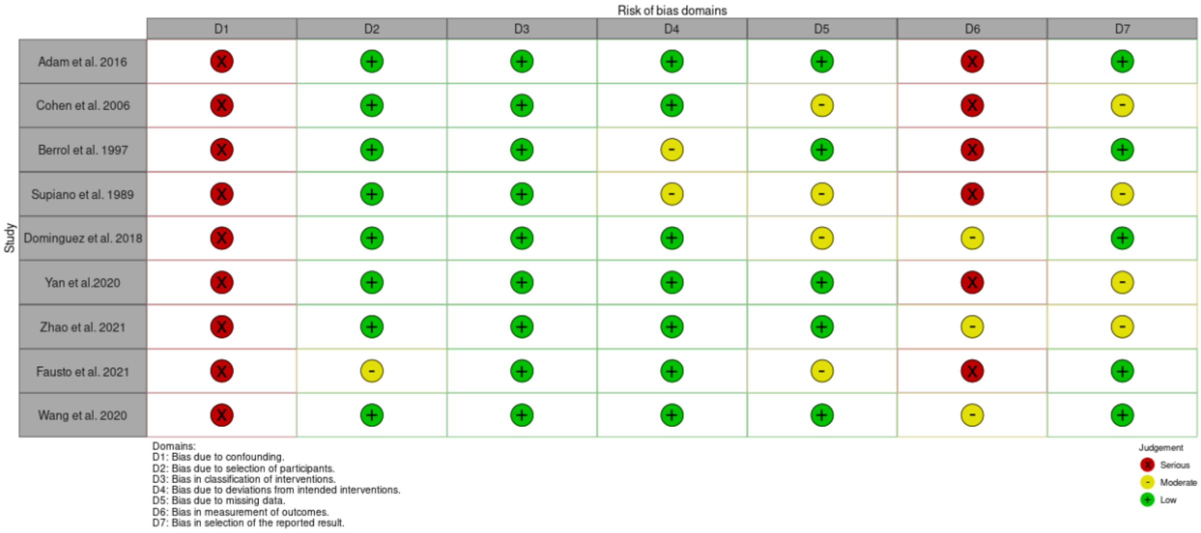

Figure 2. Traffic light plot for Risk of Bias in Non-Controlled Trials

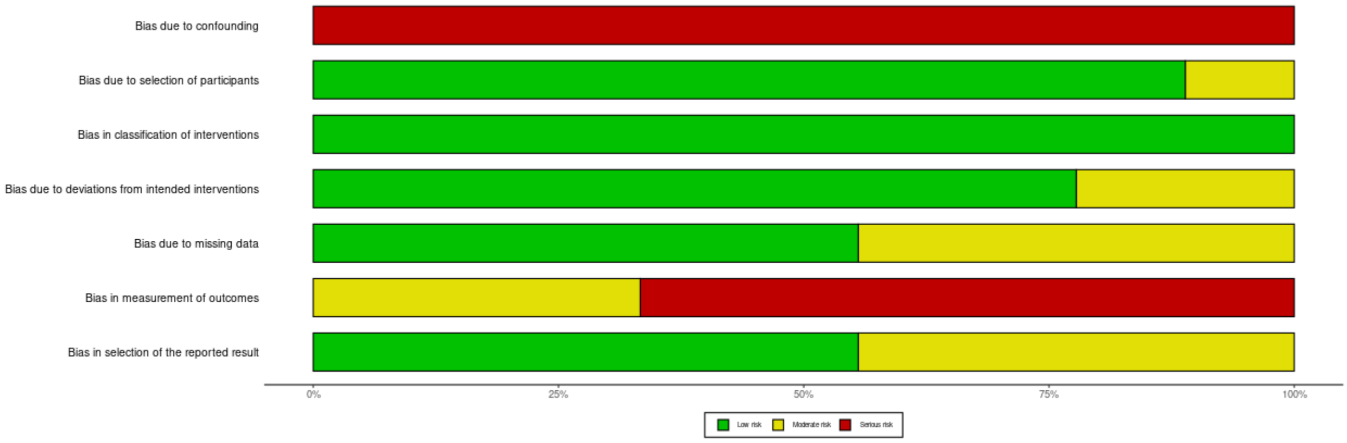

Figure 3. Summary Plot for Risk of Bias in RCTs

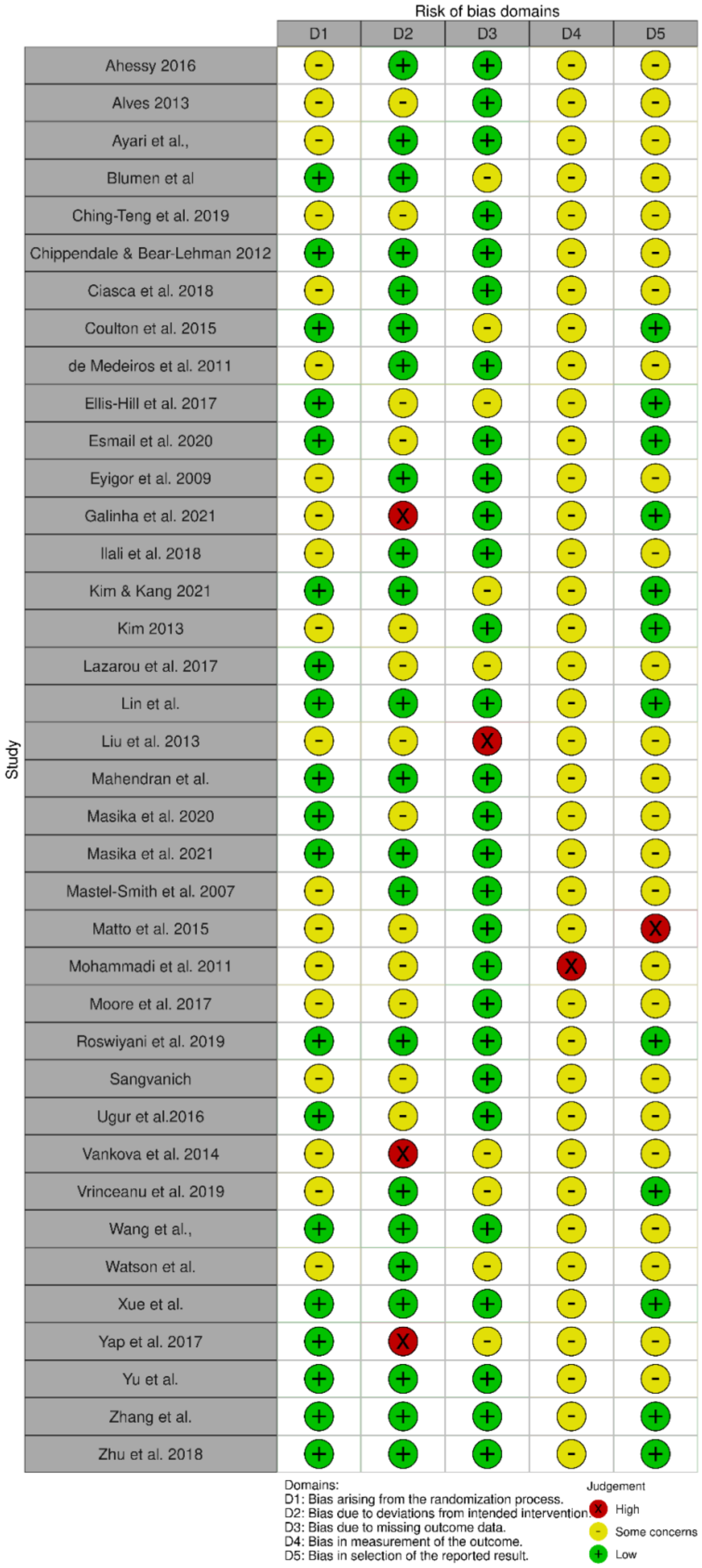

Figure 4. Traffic Light Plot for Risk of Bias in RCTs

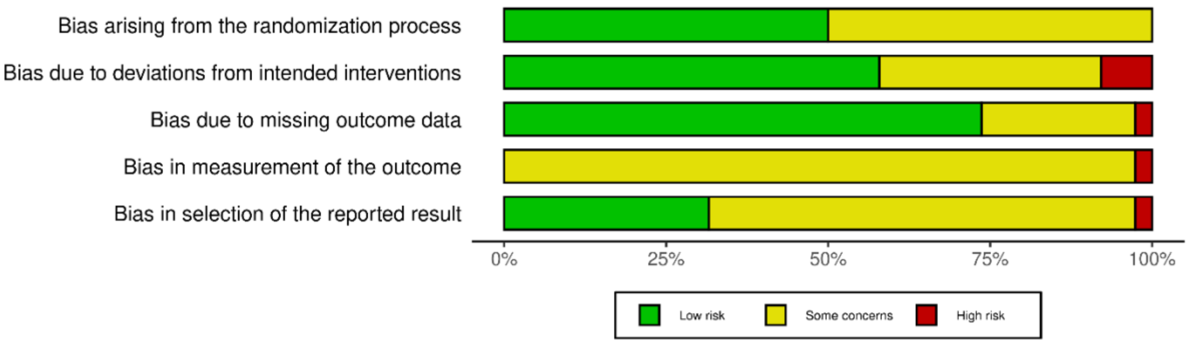

Figure 5. Summary Plot for Risk of Bias in Cluster RCTs

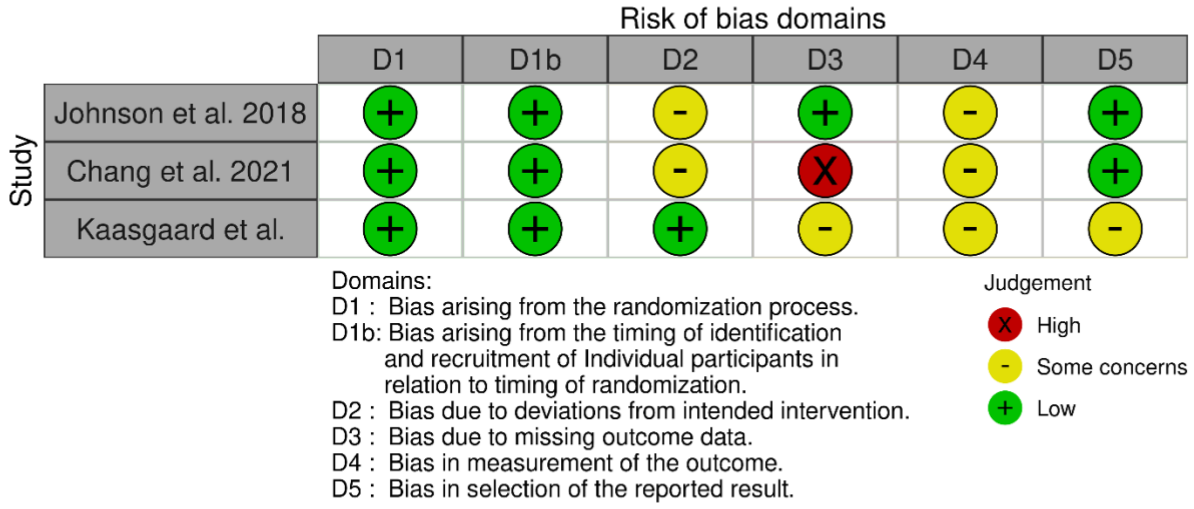

References

McGuinness, L.A., & Higgins, J.P.T. (2020). Risk-of-bias VISualization (robvis): An R package and Shiny web app for visualizing risk-of-bias assessments. *Research Synthesis Methods, 12*, 55-61. https://doi.org/10.1002/jrsm.1411

Appendix J

Prediction Interval for the effects of group arts interventions on depression

Figure 6. Distribution of true effects for depression (36 studies; *n*=3360)

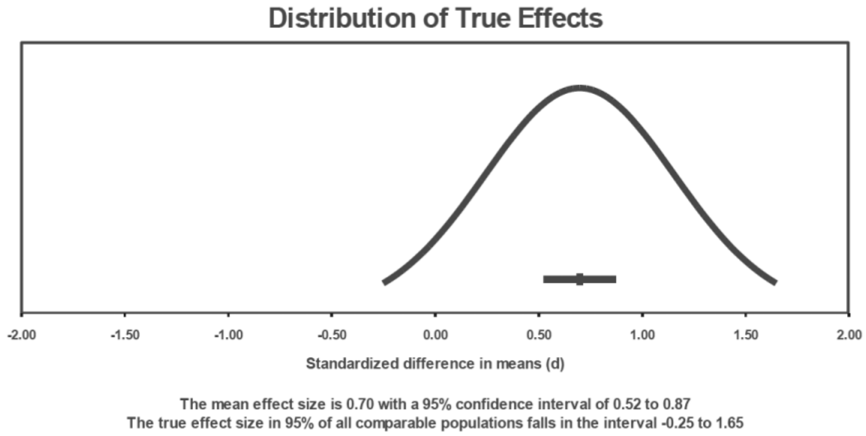

Appendix K

Prediction Interval for the effects of group arts interventions on anxiety

Figure 7. Distribution of True Effects for Anxiety (10 studies; n=949)

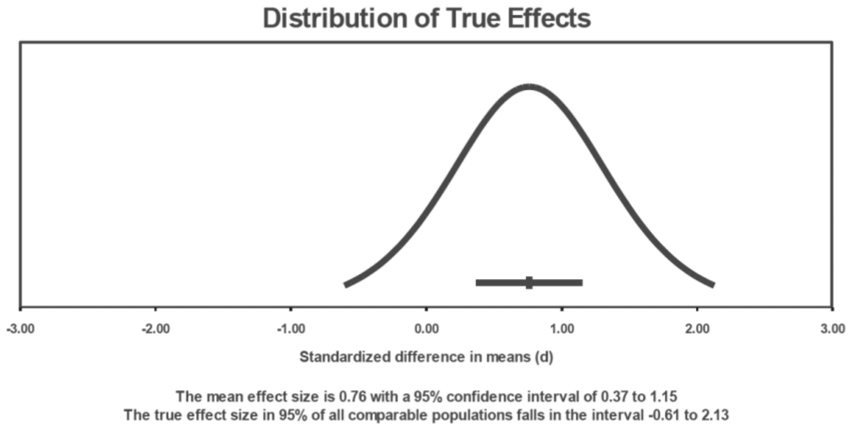

Supplement: Supplementary file 1 — Supplementary Appendixes A–K. [file 44220_2024_368_MOESM1_ESM.pdf]
