# Supplementary material for: Group arts interventions for depression and anxiety among older adults: a systematic review and meta-analysis
Source: Nat Ment Health. 2025 Mar 5;3(3):374–86. doi: 10.1038/s44220-024-00368-1 (PMC11896886; doi:10.1038/s44220-024-00368-1)
Supplement: Supplementary file 2 — Reporting Summary [file 44220_2024_368_MOESM2_ESM.pdf]

nature portfolio

Corresponding author(s): Janelle M. Jones (j.jones@qmul.ac.uk)

Last updated by author(s): Feb 18, 2025

Reporting Summary

Nature Portfolio wishes to improve the reproducibility of the work that we publish. This form provides structure for consistency and transparency in reporting. For further information on Nature Portfolio policies, see our Editorial Policies and the Editorial Policy Checklist.

Statistics

For all statistical analyses, confirm that the following items are present in the figure legend, table legend, main text, or Methods section.

n/a	Confirmed	
☐	☒	The exact sample size (*n*) for each experimental group/condition, given as a discrete number and unit of measurement
☐	☒	A statement on whether measurements were taken from distinct samples or whether the same sample was measured repeatedly
☐	☒	The statistical test(s) used AND whether they are one- or two-sided<br>*Only common tests should be described solely by name; describe more complex techniques in the Methods section.*
☐	☒	A description of all covariates tested
☐	☒	A description of any assumptions or corrections, such as tests of normality and adjustment for multiple comparisons
☐	☒	A full description of the statistical parameters including central tendency (e.g. means) or other basic estimates (e.g. regression coefficient) AND variation (e.g. standard deviation) or associated estimates of uncertainty (e.g. confidence intervals)
☐	☒	For null hypothesis testing, the test statistic (e.g. *F*, *t*, *r*) with confidence intervals, effect sizes, degrees of freedom and *P* value noted<br>*Give P values as exact values whenever suitable.*
☒	☐	For Bayesian analysis, information on the choice of priors and Markov chain Monte Carlo settings
☒	☐	For hierarchical and complex designs, identification of the appropriate level for tests and full reporting of outcomes
☐	☒	Estimates of effect sizes (e.g. Cohen's *d*, Pearson's *r*), indicating how they were calculated

Our web collection on statistics for biologists contains articles on many of the points above.

Software and code

Policy information about availability of computer code

Data collection	Publications were searched within the following databases: Cochrane Library, PsycARTICLES, PsycINFO, EMBASE, Web of Science, Pub Med and Google Scholar. Grey literature and unpublished work were sought by placing calls for literature on relevant academic listservs (European Association of Social Psychology, The Society for Personality and Social Psychology, British Society of Gerontology).<br><br>Extracted information from included studies was recorded using Microsoft Excel - Microsoft Corporation (2024). Microsoft Excel (Version 16). Microsoft Office 365.
Data analysis	The data were analysed and visualised (i.e., funnel plots, true effects) using Comprehensive Meta-Analysis (Version 3; Borenstein et al., 2013) [Computer Software]. Biostat, Englewood, NJ and the following programs and techniques:<br><br>Calculation formulae were obtained from: Borenstein, M., Cooper, H., Hedges, L., & Valentine, J. (2009). Effect sizes for continuous data. The Handbook of Research Synthesis and Meta-analysis, 2, 221-235.<br><br>Microsoft Excel was used for forest plot visualisation - Microsoft Corporation (2024). Microsoft Excel (Version 16). Microsoft Office 365.<br><br>Risk of Bias for Randomized Studies (RoB2) was calculated following: Sterne, J. A., Savović, J., Page, M. J., Elbers, R. G., Blencowe, N. S., Boutron, I., Cates, C.J., Cheng, H., Corbett, M.S., Eldridge, S.M., Emberson, J. R., Hernán, M.A., Hopewell, S., Hróbjartsson, A., Junqueira, D.R., Jüni, P., Kirkham, J.J., Lasserson, T., Tianjing, L., ... & Higgins, J. P. (2019). RoB 2: a revised tool for assessing risk of bias in randomised trials. BMJ, 366. https://doi.org/10.1136/bmj.l4898<br><br>Risk of Bias in Non-Randomised studies (ROBINS-1) was calculated following-: Sterne, J. A., Hernán, M. A., Reeves, B. C., Savović, J., Berkman,

N. D., Viswanathan, M., Henry, D., Altman, D. G., Ansari, M.T., Boutron, I., Carpenter, J.R., Chan, A., Churchill, R., Deeks, J.J., Hróbjartsson, A., Kirkham, J., Jüni, P., Loke, Y.K., Pigott, T.D., ... & Higgins, J. P. (2016). ROBINS-I: a tool for assessing risk of bias in non-randomised studies of interventions. BMJ, 355. https://doi.org/10.1136/bmj.i4919

All data, analysis code, and research materials (including our coding scheme) are available at [https://osf.io/h27ag/?view_only=6c25d5c7d93545ae83fd7cdf8d64609d]

For manuscripts utilizing custom algorithms or software that are central to the research but not yet described in published literature, software must be made available to editors and reviewers. We strongly encourage code deposition in a community repository (e.g. GitHub). See the Nature Portfolio guidelines for submitting code & software for further information.

Data

Policy information about availability of data

All manuscripts must include a data availability statement. This statement should provide the following information, where applicable:

- Accession codes, unique identifiers, or web links for publicly available datasets
- A description of any restrictions on data availability
- For clinical datasets or third party data, please ensure that the statement adheres to our policy

All data, analysis code, and research materials (including our coding scheme) are available at [https://osf.io/h27ag/?view_only=6c25d5c7d93545ae83fd7cdf8d64609d].

The following databases were searched to identify relevant articles:
Cochrane Library (Public: https://www.cochranelibrary.com)
PsycARTICLES (Institutional Access: https://www.apa.org/pubs/databases/psycarticles)
PsycINFO (Institutional Access: https://www.apa.org/pubs/databases/psycinfo)
EMBASE (Institutional Access: https://www.embase.com/)
Web of Science (Public: https://www.webofknowledge.com)
Pub Med (Public: https://pubmed.ncbi.nlm.nih.gov)
Google Scholar. (Public: https://scholar.google.com)

Human research participants

Policy information about studies involving human research participants and Sex and Gender in Research.

Reporting on sex and gender	Our 'population' are the studies included in the systematic review.The majority of studies had 75-100% female participants (n=28), or 50-74% female participants (n=15). A minority of studies had under 50% female participants (n=4). Three studies did not provide details of participants' gender.
Population characteristics	Our 'population' are the studies included in the systematic review. Of the 50 included studies, all but three studies presented the average age of their participants which ranged from 64.75 to 84.04 years. The average participant age fell into a young-old category (65-74) in more than half of studies (n=28) and into a middle-old category (75-84) in the remaining studies (n=22). As would be expected, given the longer life span of women, participants were predominantly female. Most studies did not present information about participant ethnicity or race (n=36). Of the nine studies that did include data on participant race, six had over 90% white participants. Three studies mentioned a mix of racial categories: One included 11.1% Black, 4.4% Hispanic, 9.9% Asian, and 75.6% White participants, one included 26% Non-Hispanic Black, 20% Asian, 18.5% Hispanic and 35% non-Hispanic White participants and one included 4% Native American, 24% African American, 48% White, 8% mixed race and 16% not reported. Of the five studies that included information on participants' ethnicity one had 100% African-American participants, one had 100% Korean-American participants, one had 100% Indonesian, one had 100% European-American, and one had 100% Chinese participants.
Recruitment	This project was a systematic review and meta-analysis - We did not engage in participant recruitment
Ethics oversight	Because this systematic review and meta-analysis only involved the use of secondary anonymized data from other research studies, it did not require ethical approval.

Note that full information on the approval of the study protocol must also be provided in the manuscript.

Field-specific reporting

Please select the one below that is the best fit for your research. If you are not sure, read the appropriate sections before making your selection.

☐ Life sciences ☒ Behavioural & social sciences ☐ Ecological, evolutionary & environmental sciences

For a reference copy of the document with all sections, see nature.com/documents/nr-reporting-summary-flat.pdf

Behavioural & social sciences study design

All studies must disclose on these points even when the disclosure is negative.

Study description	Systematic review and meta-analysis using subgroup and meta-regression analyses of randomised and non-randomised arts interventions reporting on depression and anxiety amongst older adults All data are quantitative.
Research sample	The studies included were published prior to February 29th, 2024. Studies were included if they reported on older adults (aged 55+) taking part in group arts interventions. Studies needed to be controlled studies (i.e., RCTs or non-randomised quasi-experimental studies with a control group) with measured of depression and/or anxiety. Studies focusing on older adults with dementia or studies reported in another language than English were excluded. We focused on older adults without dementia as they tend to be under-represented in research on the arts despite the fact that the majority of older adults do not experience dementia as a feature of aging. As we are summarising data from existing research, the sample of studies may not be representative.
Sampling strategy	Sample sizes were not predetermined as we were reporting on existing studies. Estimates derived from included studies were weighted by their standard error during meta-analysis. As a result, studies using larger samples were given more power than studies using smaller samples.
Data collection	Publications were searched within the following databases: Cochrane Library, PsycARTICLES, PsycINFO, EMBASE, Web of Science, Pub Med and Google Scholar. Grey literature and unpublished work were sought by placing calls for literature on relevant academic listservs (European Association of Social Psychology, The Society for Personality and Social Psychology, British Society of Gerontology).<br><br>Outputs were managed using Microsoft Excel (Microsoft Corporation (2024). Microsoft Excel (Version 16). Microsoft Office 365.)<br><br>10085 studies were identified in the initial search. 745 duplicates were removed from the search results. 8898 studies were excluded on title and abstract. 392 studies were excluded upon full reading (due to excluding information on the methodology, not having an arts free control group, not being group based, focusing on dementia populations, the main intervention not involving the arts, not focusing on older adults, not including depression or anxiety as outcomes). This left 50 studies to be included in the overall summary. 10 of these were not entered into the meta analysis due to a lack of data and two others were excluded as outliers, leaving 39 studies in total for the meta analysis - 36 with depression outcomes and 10 with anxiety outcomes.<br><br>There was no blinding to condition as experiments were not being run as part of this work.
Timing	Studies were identified during three searches: The initial search took place between May 8th and May 15th 2020. This search was extended between February 14th and February 28th 2022 and between February 22nd and February 29th 2024.
Data exclusions	Any arts interventions delivered on an individual basis were excluded. Any study that did not include a non-arts based control group was excluded. Studies reporting samples younger than 55 years old were excluded. Studies reporting on interventions delivered to older adults with dementia were excluded. Interventions where creative arts (e.g., music, dance, drama, writing, theatre, film) was not the main component of the intervention were excluded. Studies that did not report pre-post measures of depression and/or anxiety were excluded.
Non-participation	This meta-analysis used estimates reported by published studies, for which the participation rate was not recorded.
Randomization	All included studies were controlled interventions. Of the 50 studies entered into the summary, 8 were non-randomised. 42 were randomised.

Reporting for specific materials, systems and methods

We require information from authors about some types of materials, experimental systems and methods used in many studies. Here, indicate whether each material, system or method listed is relevant to your study. If you are not sure if a list item applies to your research, read the appropriate section before selecting a response.

Materials & experimental systems

n/a	Involved in the study	
☒	☐	Antibodies
☒	☐	Eukaryotic cell lines
☒	☐	Palaeontology and archaeology
☒	☐	Animals and other organisms
☒	☐	Clinical data
☒	☐	Dual use research of concern

Methods

n/a	Involved in the study	
☒	☐	ChIP-seq
☒	☐	Flow cytometry
☒	☐	MRI-based neuroimaging
